# Avoiding the misuse of BLUP in behavioural ecology

**DOI:** 10.1093/beheco/arx023

**Published:** 2017-03-02

**Authors:** Thomas M Houslay, Alastair J Wilson

**Affiliations:** a Centre of Ecology and Conservation, College of Life and Environmental Sciences, University of Exeter, Penryn Campus, Penryn, Conrwall, TR10 9FE, UK

**Keywords:** animal personality, behavioural plasticity, behavioural syndromes, cognition, physiology

## Abstract

Having recognized that variation around the population-level “Golden Mean” of labile traits contains biologically meaningful information, behavioural ecologists have focused increasingly on exploring the causes and consequences of individual variation in behaviour. These are exciting new directions for the field, assisted in no small part by the adoption of mixed-effects modelling techniques that enable the partitioning of among- and within-individual behavioural variation. It has become commonplace to extract predictions of individual random effects from such models for use in subsequent analyses (for example, between a personality trait and other individual traits such as cognition, physiology, or fitness-related measures). However, these predictions are made with large amounts of error that is not carried forward, rendering further tests susceptible to spurious *P* values from these individual-level point estimates. We briefly summarize the problems with such statistical methods that are used regularly by behavioural ecologists, and highlight the robust solutions that exist within the mixed model framework, providing tutorials to aid in their implementation.

## Lay Summary

Research of causes and consequences of animal personality promises exciting insights, yet widely used tests can lead to spurious results: when predictions of individual-level random effects are used in secondary analyses, their error is not carried forward, leading to increased likelihood of ‘false positive’ errors. We demonstrate how alternative approaches enable behavioural ecologists to test hypotheses about the causes and consequences of individual behavioural variation while accounting for the uncertainty inherent in the random effects.

Characterizing individual variation in behaviour is an exciting research area in behavioural ecology, with great interest in the fields of “animal personality” and individual differences in behavioural plasticity (Réale et al. 2010a; Japyassú and Malange 2014). This research is predicated on exploring previously ignored phenotypic variation: behavioural ecologists have escaped the “tyranny of the Golden Mean” in labile traits (Bennett 1987; Wilson 1998; Williams 2008), and are increasingly finding meaningful biology in what was formerly considered residual variation (Cleasby and Nakagawa 2011; Stamps et al. 2012; Brommer 2013a). Progress in these fields has been boosted by the adoption of mixed-effects modelling techniques, particularly the use of quantitative genetics-style approaches for partitioning phenotypic variation into its “between-individual” and “within-individual” components (Nussey et al. 2007; Smiseth et al. 2008; van de Pol and Wright 2009; Dingemanse et al. 2012; Dingemanse and Dochtermann 2013; Royle et al. 2014; Allegue et al. 2016). Behavioural ecologists are also increasingly interested in extending these analyses of individual behavioural variation for new avenues and purposes (Sih et al. 2004; Dall et al. 2012; Japyassú and Malange 2014; Roche et al. 2016; Stamps and Biro 2016). These typically involve exploration of the causes and consequences of individual variation in behaviour (and/or behavioural plasticity), by testing for their association with variation in other individual traits (e.g., physiological, cognitive, social, or fitness-related) or environmental variables. However, the use of anticonservative methods has become pervasive in this field. Here, we highlight not only the problems with a widely-used approach in the study of individual behavioural variation, but also the straightforward statistical solutions to these problems that should thereby hasten progress.

Specifically, it has become common practice to extract predictions of individual random effects from fitted mixed models and to use these in subsequent analyses, such as correlation tests or linear regression models (Table 1). Problems arise from this approach because individual point estimates from random effects in mixed models (sometimes known as conditional modes, or best linear unbiased predictors, BLUPs) are predicted with large amounts of error. Their use in secondary analyses can therefore lead to highly anticonservative tests of biological hypotheses, because the error inherent in their prediction is excluded from these further tests (Hadfield et al. 2010). We stress that BLUP is an incredibly useful technique that should not be dismissed in any way as inherently “bad” (Robinson 1991). Indeed, it is entirely appropriate to use individual-level predictions to say something about individuals (or genotypes, or specific levels of some other random effect). For example, scrutiny of BLUPs could be used to identify which individuals are the “boldest”, or to select individuals for groups to be used in further experimental study. However, when the objective is to say something about population-level processes or relationships then analysing sets of model predictions while ignoring their associated error is not statistically correct. This has been recognized in other fields (notably ecological and evolutionary quantitative genetics), but less so in behavioural ecology, where these improper analyses persist. As detailed by Hadfield et al. (2010), such analyses can therefore result in spuriously narrow confidence intervals and/or spuriously low *P* values that are interpreted as indicators of biological significance. While the qualitative conclusions of individual papers employing these methods may prove robust in many cases, failure to properly account for uncertainty will increase Type I errors (false positives) across the field. In short, published *P* values are systematically anticonservative and should not be taken at face value.

**Table 1:** Examples in the behavioural literature of questions regarding individual variation in behaviour (“personality”) and behavioural plasticity, using best linear unbiased predictors (BLUPs) in secondary analyses rather than multivariate models

Test	Species	Reference
Behavioural syndromes	*Microtus arvalis*	(Lantová et al. 2011)
	*Taeniopygia guttata*	(Wuerz and Krüger 2015)
	*Latrodectus hesperus*	(Montiglio and DiRienzo 2016)
Personality across life stages	*Tamiasciurus hudsonicus*	(Kelley et al. 2015)
Different measures of a single personality trait	*Bitis arietans*	(Carter et al. 2012c)
	*Pomacentrus wardi, P. amboinensis*	(Beckmann and Biro 2013)
Personality and sampling bias	*Agama planiceps*	(Carter et al. 2012b)
Personality and hormones	*Tamias striatus*	(Montiglio et al. 2012)
	*Canis latrans*	(Schell et al. 2016)
Personality and physiology	*Cavia aperea*	(Guenther and Trillmich 2015)
	*C. aperea*	(Finkemeier et al. 2016)
Personality and telomere length	*Salmo trutta*	(Adriaenssens et al. 2016)
Personality and cognition	*C. aperea*	(Guenther et al. 2014)
	*C. aperea, C. porcellus*	(Brust and Guenther 2015)
Personality and social network attributes	*Anguilla anguilla*	(Geffroy et al. 2014)
	*Marmota flaviventris*	(Fuong et al. 2015)
Personality and local density	*T. hudsonicus*	(Shonfield et al. 2012)
Personality and social niche specialisation	*Suricata suricatta*	(Carter et al. 2014)
Personality and group-size preference	*Perca fluviatilis*	(Hellström et al. 2016)
Personality and predation risk	*P. fluviatilis*	(Magnhagen et al. 2012)
		(Heynen et al. 2016)
Personality and mating behaviour	*Aquarius remigis*	(Wey et al. 2014; Wey et al. 2015)
	*Gerris buenoi*	(Pineaux and Turgeon 2017)
Personality and breeding performance	*Circus pygargus*	(Arroyo et al. 2017)
Personality and survival	*T. striatus*	(Bergeron et al. 2013)
Personality and fitness-related traits	*S. trutta*	(Adriaenssens and Johnsson 2011)
Personality and individual variation in behavioural plasticity	*A. planiceps*	(Carter et al. 2012a)
	*Microcebus murinus*	(Dammhahn and Almeling 2012)
	*T. guttata*	(Gibelli and Dubois 2016)
Personality, behavioural plasticity, and reproductive success	*Tachycineta bicolor*	(Betini and Norris 2012)
Personality, behavioural plasticity, and mating	*A. remigis*	(Montiglio et al. 2016a; Montiglio et al. 2016b)
Personality, behavioural plasticity, and fitness	*Tenagogerris euphrosyne*	(Han and Brooks 2014)

All were published after the publication of Hadfield *et al* (2010).

Recent examples of publications (mis-)using BLUPs include tests of associations between personality (and/or individual variation in behavioural plasticity) and a wide range of traits, including physiology, cognition, social networks, niche specialization, and fitness (Table 1). In many cases, authors have explicitly acknowledged the potential for problems as outlined by Hadfield et al. (2010). Nonetheless, use of these “stats on stats” approaches that are known to be inappropriate for hypothesis testing (see Brommer 2013b for further discussion) continues unabated. This is presumably because researchers are not aware of how to implement more robust analytical strategies, and/or because of a misconception that problems are restricted to quantitative genetic models. On the latter point we note that predictions from mixed models in which random effects are assumed to covary between individuals (through e.g., genetic relatedness, spatial/temporal autocorrelation, or social processes) cannot be treated as independent “data points”. However, this in no way justifies ignoring uncertainty when random effects are predicted from a model that assumes no among-individual covariance.

**Figure 1:**
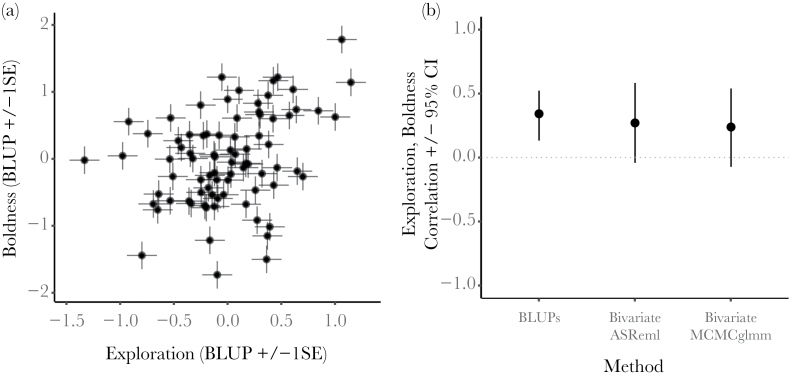
Taken from a worked example provided in the Supplementary Information, (a) shows a scatterplot of individual-level estimates (BLUPs) of 2 personality traits, extracted from separate univariate models. Bars around each point show the standard error of the estimate for both traits, which is ignored by subsequent analyses of these BLUPs. Testing a correlation using only BLUPs and ignoring their error results in an anticonservative test, as illustrated in (b). The correlation test using BLUPs produces narrow confidence intervals, and a correspondingly small *P* value of 0.0019, indicating statistical significance (“BLUP” on *x* axis). However, testing the correlation directly in a bivariate model using REML and retaining all data returns larger (approximate) confidence intervals which straddle zero (95% CI approximated as *r* ± 1.96SE) and a *P* value (based on a likelihood ratio test) of 0.12, such that the correlation is not statistically significant (“Bivariate ASReml” on *x* axis). Using the same data, Bayesian 95% credible intervals also cross zero, which indicates a lack of statistical significance (“Bivariate MCMCglmm").

Fortunately, the mixed-effects model framework does offer a way to test hypotheses such as those listed above while fully accounting for the uncertainty inherent in the random effects. An overreliance on the (otherwise excellent) lme4 package for mixed models in R (Bates et al. 2015) may have held many behavioural ecologists in the “Flatland” of univariate modelling (Walsh 2007). In the majority of cases, questions that are multivariate in nature are best answered using a multivariate framework. That is, a modelling framework containing multiple response variables, enabling 1) testing of how explanatory variables (“fixed effects”) predict these responses, as in standard univariate models, and 2) the simultaneous estimation of the variance of each response and the covariance between them, at group levels specified within the random effects structure. It is relatively straightforward to rephrase these multivariate questions in terms of variances and covariances (or derived correlations and regressions), and to fit multivariate models accordingly (some examples include Ferrari et al. 2013; Kluen et al. 2013; Royauté et al. 2013; Boulton et al. 2014; Careau et al. 2015; Niemelä et al. 2015; Petelle et al. 2015; Sanderson et al. 2015; Santostefano et al. 2016; Vallon et al. 2016; White et al. 2016). For instance, we might hypothesize a behavioural syndrome in which positive correlations are predicted between the (repeatable) tendencies of individuals to exhibit 3 behaviours. Having assayed each of these behaviours on multiple occasions for a set of individuals, the correct approach would be to estimate—and test the significance of—those among-individual correlations directly in a trivariate mixed model incorporating all of the behavioural data. This method yields correlation estimates with valid measures of uncertainty (SE or CI). This is not the case when generating individual-level random effect predictions from 3 separate univariate models (one for each behaviour) and then testing whether they are correlated. In the latter approach, uncertainty will be underestimated and thus Type I error is more likely to occur (Figure 1).

On a pragmatic point, we note that it is not required that each variable of interest be a repeated measure in these models—for example, it is perfectly feasible to test for the existence of an among-individual correlation between a personality trait (with repeated measures) and some other variable with only one observation per individual, such as an estimate of its lifetime reproductive success. In the Supplementary Material, we provide worked examples of how to set up multivariate statistical models to address these (and several similar) questions using the R packages ASReml-R (Butler 2009) and MCMCglmm (Hadfield 2010). These examples provide users with the tools to test their hypotheses in a multivariate framework, incorporating all of their data and avoiding potentially spurious results.

We also note that multivariate mixed models may often provide a more appropriate route to testing hypotheses about multivariate phenotypes in other contexts. For instance, one approach to exploring behavioural syndromes has been to reduce the dimensionality of observed behaviours by performing principal components analysis (PCA) on multivariate data, and then to use univariate mixed models to calculate repeatability on individual scores for each component (e.g., Edenbrow and Croft 2013; Le Galliard et al. 2013; Brent et al. 2014; Patrick and Weimerskirch 2014; Sussman et al. 2014; Rangassamy et al. 2015). This allows us to ask whether, for instance, the major axis of observed behavioural (co)variation is repeatable. This is a valid question but in many cases perhaps not the most pertinent one, since the first principal component of observed variation includes both among- and within-individual trait variation. For studies of individual differences in behaviour, the more relevant question might be better focused at the among-individual level—that is, what does the major axis of among-individual variation look like? If so, then isolating the among-individual (co)variance matrix (sometimes denoted **I**; Wilson et al. 2011) by applying a multivariate mixed model to a set of traits is the proper first step. Principal components (or eigenvectors) of **I** can then be examined directly. This strategy is probably more appropriate for testing models such as “pace of life syndrome” or stress coping styles that posit trait correlations at the among-individual level—i.e., that these correlations are due to consistent differences among individuals, and not because of some temporary aspect of environmental variation (Koolhaas et al. 2007; Carere et al. 2010; Coppens et al. 2010; Réale et al. 2010b; Carter et al. 2013). The value of partitioning individual (co)variances has been discussed in more detail by Brommer (2013a), and illustrations exist in the literature of the use of multivariate mixed models for studying pace of life syndrome (White et al. 2016) and stress coping styles (Boulton et al. 2015).

We fully acknowledge that multivariate mixed models are data hungry. However, a failure of these multivariate models to converge to sensible and/or precise solutions does not mean that we can retreat to the relative comfort of previous methods: in fact, this is likely to indicate a lack of power to answer the question at hand (see Martin et al. 2011; Wolak et al. 2012). In cases where logistical constraints prevent there being enough measurements to partition out the among-individual behavioural (co)variation, a preferable method may sometimes be to work with observed phenotypic (co)variance while acknowledging this and the assumptions that underpin conclusions drawn. Indeed, much of behavioural ecology is predicated on the “phenotypic gambit”, the assumption that phenotypic patterns of trait (co)variation (denoted **P**) provide a workable proxy for patterns of genetic (co)variance (**G**). If **P** can be used (with caveats) in place of **G** where estimation of genetic parameters is not feasible, then it can also be used (with caveats) in place of **I** where partitioning of among-individual covariation from within-individual covariation is not feasible.

To conclude, we absolutely wish to encourage more studies that further our understanding of the causes and consequences of individual differences in behaviour. However, we also make a plea to the community to avoid inappropriate methods of analysis that lead to spurious precision and increased Type I errors. This field depends upon embracing the power of previously ignored phenotypic variation, and it is flourishing because of the exciting questions we can now address—but we must ensure that we use the right tools when doing so.

## SUPPLEMENTARY MATERIAL

Supplementary data are available at *Behavioral Ecology* online.

## FUNDING

This work was supported by a Biotechnology and Biological Sciences Research Council grant (BB/L022656/1) awarded to A.J.W.

## Supplementary Material

IndivVar_MV_tutorial_MCMCglmm

syndrome

IndivVar_MV_tutorial_ASReml
