# Supplementary material for: Avoiding the misuse of BLUP in behavioural ecology
Source: Behav Ecol. 2017 Mar 2;28(4):948–52. doi: 10.1093/beheco/arx023 (PMC5873244; doi:10.1093/beheco/arx023)
Supplement: IndivVar_MV_tutorial_MCMCglmm [file arx023_suppl_indivvar_mv_tutorial_mcmcglmm.pdf]

Avoiding the misuse of BLUP in behavioral ecology: I. Multivariate modelling for individual variation (MCMCglmm tutorial)

T.M. Houslay & A.J. Wilson, Behavioral Ecology

January 2017

Introduction

Overview

This tutorial accompanies our 2017 Behavioral Ecology paper, "Avoiding the misuse of BLUP in behavioral ecology". Below, we provide worked examples of multivariate statistical methods for directly testing hypotheses about associations between individual variation in behaviour and other traits. Below, we will:

- Test the correlation between two personality traits (behaviours measured repeatedly on individuals);
- Test for an association between these personality traits and a measure of fitness (one value per individual).

In this version of the tutorial, we illustrate these models using the `R` package `MCMCglmm`, developed by Jarrod Hadfield. Visit the CRAN page for `MCMCglmm` here for links and citation info: https://cran.r-project.org/web/packages/MCMCglmm/index.html.

MCMCglmm fits generalised linear mixed modes (GLMMs) in a Bayesian framework, using Markov chain Monte Carlo techniques. We have also provided a separate tutorial for the `R` interface for `ASReml`, which fits GLMMs using maximum likelihood (and so is likely more familiar to `lme4` users) but is commercially licensed software.

Updates and further tutorials associated with this paper can be found at https://tomhouslay.com/tutorials/.

Aims

Please note that we do assume readers are familiar with the general principles of specifying mixed effects models, and in particular with the use of `MCMCglmm` for univariate mixed effects models. Readers unfamiliar with using univariate mixed effects models for modelling a single behavioural trait might prefer to start with Dingemanse & Dochtermann's 2013 paper, 'Quantifying individual variation in behaviour: mixed effects modelling approaches'. Readers unfamiliar with `MCMCglmm` should look at Jarrod Hadfield's excellent course notes, available at the `MCMCglmm` CRAN page: https://cran.r-project.org/web/packages/MCMCglmm/index.html.

We also use various methods for manipulating and visualising data frames using the `tidyverse` package (including `tidyr`, `dplyr`, `ggplot2` etc) — more details on their use can be found at http://r4ds.had.co.nz/.

In our tutorial, we aim to teach the following:

- How to phrase questions of interest in terms of variances and covariances (or derived correlations or regressions);
- How to incorporate more advanced model structures, such as:
- Fixed effects that apply only to a subset of the response traits;
- Traits which are measured a different number of times (*e.g.*, repeated measures of behaviour and a single value of breeding success);
- Interpreting MCMC credible intervals.

Packages required

There are several packages that you must have installed in `R` prior to starting this tutorial:

- `MCMCglmm`
- `lme4`
- `nadiv`
- `tidyverse`
- `broom`

'Study system'

For this tutorial, we have collected data on populations of wild haggis that roam the Highlands of Scotland.

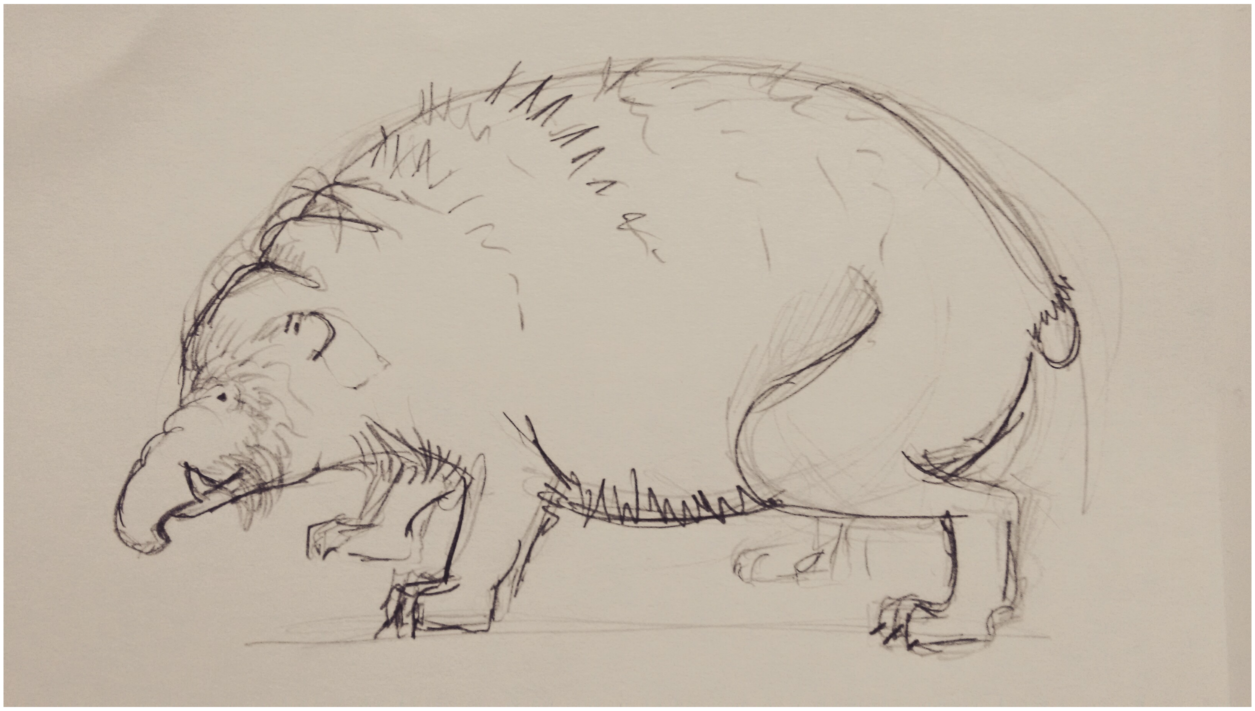

Figure 1: A male haggis in the wild (thanks to Emma Wood, http://www.ewood-art.co.uk/)

We tag all haggis individually when they emerge from their burrows as juveniles in their first spring. Here, we concentrate on male haggis, which are solitary and territorial. Previous work has identified behaviours that can be measured repeatedly, and used to represent three personality traits: **boldness**, **exploration**, and **aggression**. We also have the ability to collect a single measure of mating success (as a fitness proxy) for each male at the end of the season.

Behavioural syndromes

One type of 'behavioural syndrome' is a correlation between personality traits. Since personality can be viewed (under most definitions) as the repeatable (among-individual) component of behaviour, evidence for the presence of a behavioural syndrome is provided by covariance among behaviours that arises from among-individual differences.

Here we have repeatedly measured behaviours that represent **boldness** and **exploration**. We observed each behaviour 4 times per individual. We also measured their body size on the day of behavioural assay to control for general size effects. in our statistical models.

Load libraries and inspect data

```
library(lme4)
library(MCMCglmm)
library(tidyverse)
library(broom)
library(nadiv)

df_syndrome <- read_csv("syndrome.csv")
```

This data frame has 6 variables:

- Individual **ID**
- The repeat number for each behavioural test, **assay_rep**
- **boldness**, measured 4 times per individual
- **exploration**, measured 4 times per individual
- **fitness**, our measure of mating success, with a single value for each individual
- Individual **body_size**, as measured on the day of testing.

Univariate models

We first use the `R` package `lme4` to determine the proportion of phenotypic variation (adjusted for fixed effects) that is due to differences among individuals, separately for each behaviour. We assume readers have knowledge of these 'univariate' models and their use in behavioural studies — if not, there are various other publications that go into them in greater detail (e.g., Dingemanse & Dochtermann (2013)).

Boldness

Our model includes fixed effects of the assay repeat number (centred) and individual body size (centred and scaled to standard deviation units), as we wish to control for any systematic effects of these variables on individual behaviour. Please be aware that controlling variables are at your discretion — for example, while we want to characterise among-individual variance in boldness after controlling for size effects in this study, others may wish to characterise among-individual variance in boldness without such control. Indeed, using the techniques shown later in this tutorial, it would be entirely possible to characterise both among-individual variance in boldness and in size, and the among-individual covariance between these measurements.

```
lmer_b <- lmer(boldness ~ scale(assay_rep, scale=FALSE) +
                 scale(body_size) +
                 (1|ID),
               data = df_syndrome)
plot(lmer_b)
qqnorm(residuals(lmer_b))
hist(residuals(lmer_b))

summary(lmer_b)
```

```
## Linear mixed model fit by REML ['lmerMod']
## Formula: boldness ~ scale(assay_rep, scale = FALSE) + scale(body_size) +
##     (1 | ID)
##    Data: df_syndrome
##
## REML criterion at convergence: 1061.4
##
## Scaled residuals:
##     Min      1Q  Median      3Q     Max
## -2.3645 -0.6496 -0.1154  0.6463  2.6894
##
## Random effects:
##  Groups   Name        Variance Std.Dev.
##  ID       (Intercept) 0.6951   0.8337
##  Residual             1.1682   1.0808
## Number of obs: 320, groups:  ID, 80
##
## Fixed effects:
##                                 Estimate Std. Error t value
## (Intercept)                     20.09133    0.11108  180.87
## scale(assay_rep, scale = FALSE) -0.04805    0.05404   -0.89
## scale(body_size)                 0.14128    0.10893    1.30
##
## Correlation of Fixed Effects:
##             (Intr) s(_s=F
## s(_,s=FALSE  0.000
## scl(bdy_sz)  0.000 -0.002
```

Having examined diagnostic plots of the model fit, we can check the model `summary`. We are interested in the *random effects* section of the `lme4` model output (specifically the **variance** component — note that the standard deviation here is simply the square root of the variance). Evidence for 'animal personality' (or 'consistent among-individual differences in behaviour') in the literature is largely taken from the **repeatability** of behaviorual traits: we can compute this **repeatability** (also known as the *intraclass correlation coefficient*) by dividing the variance in the trait due to differences among individuals (V_{ID}) by the total phenotypic variance after accounting for the fixed effects ($V_{ID} + V_{residual}$). This can be done quickly and automatically through the use of the R package `broom`:

```
rep_bold <- tidy(lmer_b, effects = "ran_pars", scales = "vcov") %>%
  select(group, estimate) %>%
  spread(group, estimate) %>%
  mutate(repeatability = ID/(ID + Residual))

rep_bold
```

ID	Residual	repeatability
0.695	1.168	0.373

So we can see that 37.3% of the phenotypic variation in boldness (having controlled for body size and assay repeat number) is due to differences among individuals.

Let's do the same for our other behavioural trait, exploration:

Exploration

```
lmer_e <- lmer(exploration ~ scale(assay_rep, scale=FALSE) +
                 scale(body_size) +
                 (1|ID),
               data = df_syndrome)
```

```
rep_expl <- tidy(lmer_e, effects = "ran_pars", scales = "vcov") %>%
  select(group, estimate) %>%
  spread(group, estimate) %>%
  mutate(repeatability = ID/(ID + Residual))
```

ID	Residual	repeatability
0.362	0.909	0.285

Both of our traits of interest are repeatable at the among-individual level — the remaining question is characterising the association between these personality traits. Are individuals that are consistently bolder than average also more exploratory than average (and vice versa)?

Correlation using BLUPs

In our paper, we advise against the use of BLUPs due to their potential for spurious results due to anticonservative hypothesis tests and/or confidence intervals.

Here we will run through this method, purely so that we can then contrast the results with those that we get having (correctly) estimated the among-individual correlation between these behaviours directly from a multivariate model (in this case, bivariate).

We create two data frames of individual predictions extracted from model fits, one for each of our univariate `lme4` models for boldness and exploration. We then join these (by individual ID) to create a single data frame:

```
df_BLUPS_B <- data_frame(ID = row.names(ranef(lmer_b)$ID),
                         BLUP_B = ranef(lmer_b)$ID[,"(Intercept)"])

df_BLUPS_E <- data_frame(ID = row.names(ranef(lmer_e)$ID),
                         BLUP_E = ranef(lmer_e)$ID[,"(Intercept)"])

df_BLUPS_EB <- left_join(df_BLUPS_E,
                         df_BLUPS_B,
                         by = "ID")
```

We can plot these to see what our expectation of a correlation might be:

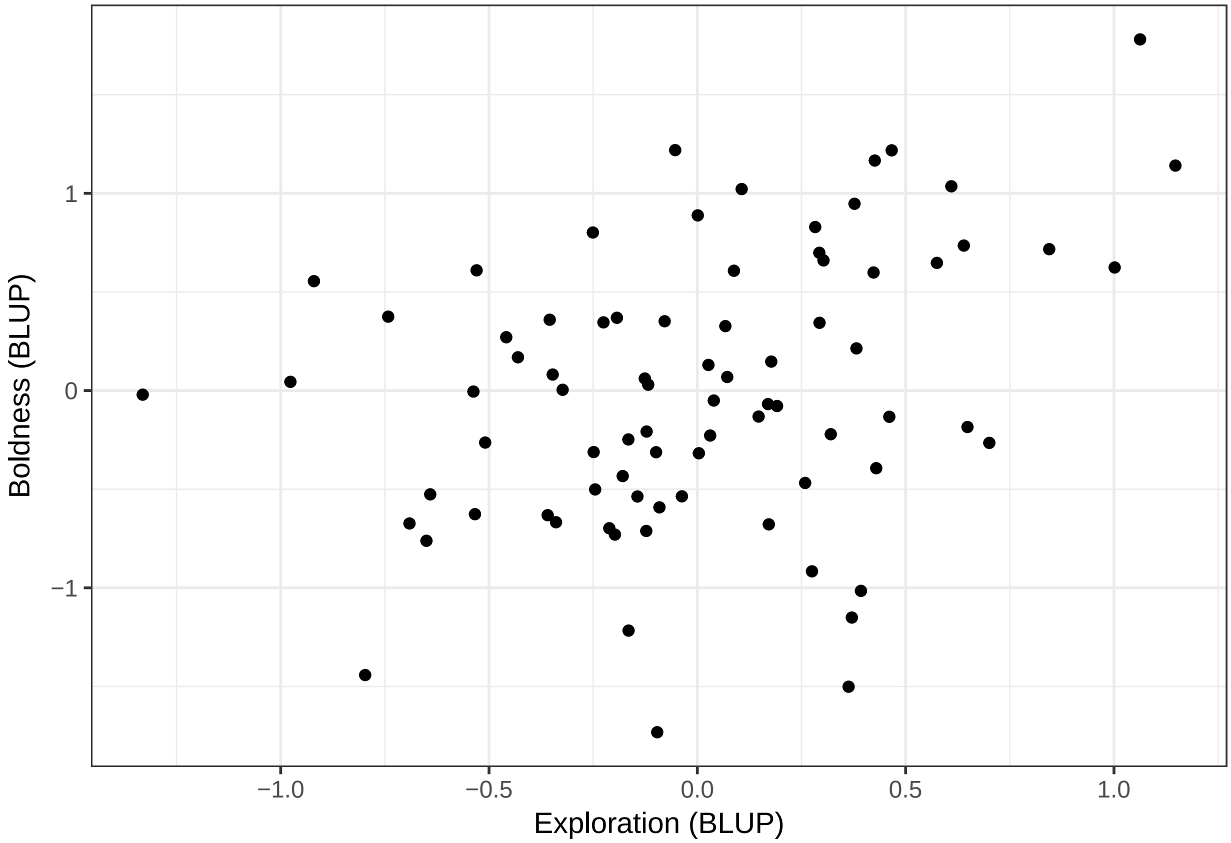

..and then simply perform a correlation test of these two traits using the `cor.test` function:

```
cor.test(df_BLUPS_EB$BLUP_E,
         df_BLUPS_EB$BLUP_B)
```

```
##
##  Pearson's product-moment correlation
##
## data:  df_BLUPS_EB$BLUP_E and df_BLUPS_EB$BLUP_B
## t = 3.2131, df = 78, p-value = 0.00191
## alternative hypothesis: true correlation is not equal to 0
## 95 percent confidence interval:
##  0.1320924 0.5223645
## sample estimates:
##       cor
## 0.3418867
```

As you can see, we get a positive correlation with a very small p-value ($P = 0.0019$), indicating that these traits are involved in a behavioural syndrome. While the correlation itself is fairly weak ($r = 0.34$), it appears to be highly significant, and suggests that individuals that are bolder than average also tend to be more exploratory than average.

However, as discussed in our paper (and in greater detail by Hadfield *et al*), using BLUPs in this way leads to anticonservative significance tests. This is because the error inherent in their prediction is not carried forward from the `lmer` models to the subsequent analysis (in this case, a correlation test). To illustrate this point quickly, below we plot the individual estimates along with their associated standard errors:

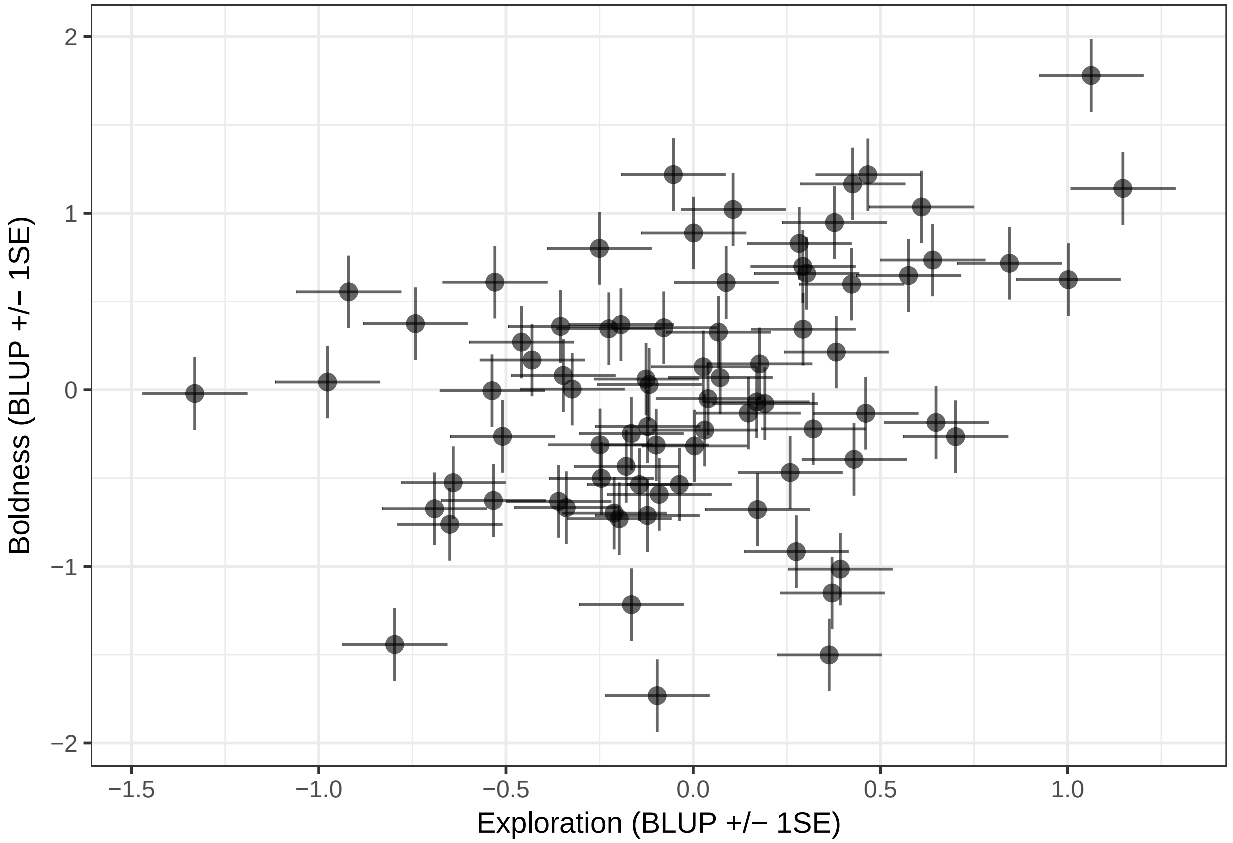

We now go on to estimate the correlation between these behaviours directly in a multivariate model, using `MCMCglmm`.

Bivariate models

The correct approach for testing the hypothesised behavioural syndrome uses both response variables in a two-trait ('bivariate') mixed model. This model estimates the among-individual variance for each response variable (and the covariance between them). Separate (co)variances are also fitted for the residual variation. The bivariate model also allows for fixed effects to be fitted on both response variables.

First, we need to create a 'prior' for our model. We recommend reading up on the use of priors; briefly, we use a parameter-expanded prior here that should be uninformative for our model. One of the model diagnostic steps that should be used later is to check that the model is robust to multiple prior specifications.

```
prior_E_B_1px = list(R = list(V = diag(2), nu = 0.002),
                     G = list(G1 = list(V = diag(2), nu = 2,
                                        alpha.mu = rep(0,2),
                                        alpha.V = diag(25^2,2,2))))
```

We set up our model using the `MCMCglmm` function call, with our bivariate response variable being **exploration** and **boldness** bound together using `cbind`. You will also note that we `scale` our response variables, meaning that each is centred at their mean value and standardised to units of 1 phenotypic standard deviation. This simply makes it easier for the model to be fit, and for us to understand the output, as both boldness and exploration are now on the same scale.

```
mcmc_E_B_us <- MCMCglmm(cbind(scale(exploration), scale(boldness)) ~ trait-1 +
                          trait:scale(assay_rep, scale = FALSE) +
                          trait:scale(body_size),
                        random =~  us(trait):ID,
                        rcov =~ us(trait):units,
                        family = c("gaussian","gaussian"),
                        prior = prior_E_B_1px,
                        nitt=420000,
                        burnin=20000,
                        thin=100,
                        verbose = TRUE,
                        data = as.data.frame(df_syndrome))
```

On the right hand side of our model formula, we use the `trait` keyword to specify that this is a multivariate model — `trait-1` effectively tells the model to give us a distinct intercept for each trait. We then interact `trait` with our fixed effects, **assay_rep** and **body_size**, so that we get estimates for the effect of these variables on each of our behaviours.

Our random effects structure starts with the `random` effects, where we tell the model to fit an 'unstructured' (`us`) covariance matrix for the grouping variable **ID**. This means that we want to calculate the variance in exploration due to differences among individuals, the variance in boldness due to differences among individuals, and the **covariance** between these variances.

Next, we set a structure for the residual variation (`rcov`), which is also sometimes known as the 'within-individual variation'. As we have repeated measures for both traits at the individual level, we also set an unstructured covariance matrix, which finds the residual variance for each trait and also allows these variances to covary.

We then provide the name of the object we set up as the model prior, and values for the total number of iterations (`nitt`), the 'burn-in' of initial iterations to be discarded as the model starts to converge (`burnin`), and the number of iterations to discard in between successive stored samplles (`thin`, which helps to reduce autocorrelation in sampling).

Finally, we provide the name of the data frame — we enclose this in the `as.data.frame` function as `MCMCglmm` does not work with the `tbl_df` format used in the **tidyverse** group of packages.

After the model has been fit by `MCMCglmm` (which will take some time!), we can check some model diagnostics using plots of the MCMC samples. Here we show just the plots for our variance components (these plots are also available for fixed effects, using `Sol`):

```
plot(mcmc_E_B_us$VCV)
```

For current purposes these should look fine, assuming you have used our simulated data and the settings above. Note however that for any real analysis various other tests (e.g. of autocorrelation, robustness to different priors, and good model convergence using the `geweke.diag` and `gelman.diag` diagnostic functions) should be used before accepting final results.

The `summary` part of the `MCMCglmm` model fit contains a large amount of information. Some general information at the start of the summary includes the model DIC. The `G-structure` then contains information about the random effects (co)variances, the `R-structure` the residual (co)variances, and the `Location effects` holds the fixed effects results information.

Each of these sections provides the mean of the posterior distribution returned by `MCMCglmm`, in addition to the lower and upper bounds of the 95% credible intervals. The effective sample size is also provided, and – for the fixed effects only – a pMCMC value.

```r
summary(mcmc_E_B_us)
```

```
##
##  Iterations = 20001:419901
##  Thinning interval  = 100
##  Sample size  = 4000
##
##  DIC: 1596.616
##
##  G-structure:  ~us(trait):ID
##
##                                       post.mean l-95% CI u-95% CI eff.samp
## traitexploration:traitexploration.ID   0.29234  0.14609   0.4538     4000
## traitboldness:traitexploration.ID      0.08287 -0.03125   0.2079     4000
## traitexploration:traitboldness.ID      0.08287 -0.03125   0.2079     4000
## traitboldness:traitboldness.ID         0.38889  0.22405   0.5735     4000
##
##  R-structure:  ~us(trait):units
##
##                                          post.mean l-95% CI u-95% CI
## traitexploration:traitexploration.units    0.7340   0.5996   0.8697
## traitboldness:traitexploration.units       0.3338   0.2390   0.4353
## traitexploration:traitboldness.units       0.3338   0.2390   0.4353
## traitboldness:traitboldness.units          0.6391   0.5287   0.7614
##                                          eff.samp
## traitexploration:traitexploration.units     4000
## traitboldness:traitexploration.units        3365
## traitexploration:traitboldness.units        3365
## traitboldness:traitboldness.units           3685
##
##  Location effects: cbind(scale(exploration), scale(boldness)) ~ trait - 1 + trait:scale(assay_rep, s
##
##                                                    post.mean   l-95% CI
## traitexploration                                   0.0002371 -0.1503944
## traitboldness                                     -0.0013789 -0.1529724
## traitexploration:scale(assay_rep, scale = FALSE) -0.0226367 -0.1030113
## traitboldness:scale(assay_rep, scale = FALSE)    -0.0355084 -0.1083371
## traitexploration:scale(body_size)                  0.0714747 -0.0887465
## traitboldness:scale(body_size)                     0.1047925 -0.0543119
##                                                     u-95% CI eff.samp pMCMC
## traitexploration                                   0.1557892     4000 0.992
## traitboldness                                      0.1667160     4000 0.992
## traitexploration:scale(assay_rep, scale = FALSE)  0.0599347     4000 0.586
## traitboldness:scale(assay_rep, scale = FALSE)     0.0473711     4000 0.392
## traitexploration:scale(body_size)                  0.2192468     3779 0.349
## traitboldness:scale(body_size)                     0.2627610     4000 0.184
```

Note that you will **not** have exactly the same results as we have, because of the way that the MCMC process works — if you run it again yourself, you will get slightly different answers again. However, they should be very similar.

From the fixed effects, we can see that there is a separate intercept for both personality traits (no surprise that these are very close to zero, given that we mean-centred and scaled each trait before fitting the model),

and an estimate of the effect of assay repeat and body size on both traits. None of these appear to be large effects, so let's move on to the more interesting parts — the random effects estimates.

In the `G-structure`, we have the among-individual (co)variances. These are given such that they can be reformed into a matrix, which is why $V_{boldness}$ and $V_{exploration}$ are shown once each, while the among-individual covariance between them ($COV_{boldness,exploration}$) is shown twice.

You will notice that the variance estimates here are actually close to the `lme4` repeatability estimates, which is because we scaled our response variables to phenotypic standard deviations. We can also find the 'adjusted repeatability' (i.e., the repeatability conditional on the fixed effects) for each trait by dividing its among-individual variance estimate by the sum of its among-individual and residual variances. To do this, we can create a new posterior distribution of (for example) 'proportion of exploration variance explained by differences among individuals'. We do this by referencing the different variance components by their name as shown in the summary (note that sometimes different versions display these with or without the 'trait' prefix, so check how yours has displayed).

```
mcmc_prop_E <- mcmc_E_B_us$VCV[,"traitexploration:traitexploration.ID"]/(
  mcmc_E_B_us$VCV[,"traitexploration:traitexploration.ID"] +
    mcmc_E_B_us$VCV[,"traitexploration:traitexploration.units"]
)

plot(mcmc_prop_E)
```

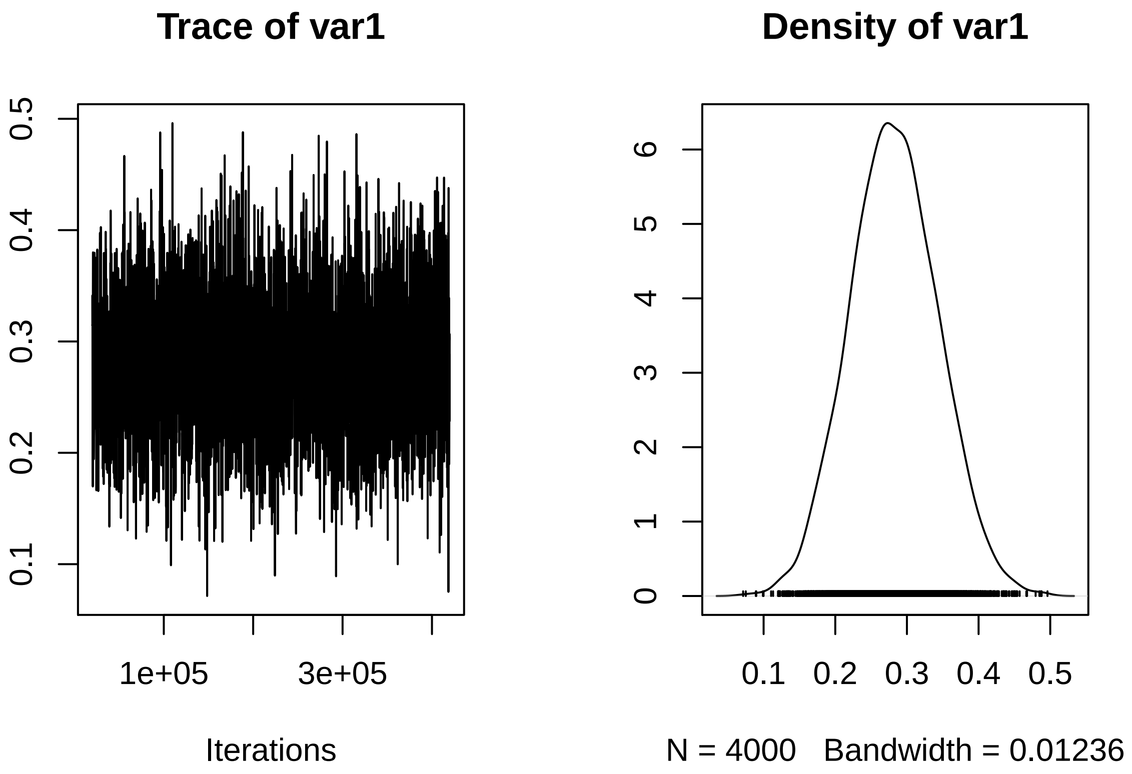

We can interrogate this new distribution for its mean and 95% CIs:

```
mean(mcmc_prop_E)
```

```
## [1] 0.2824676
```

```
HPDinterval(mcmc_prop_E)
```

```
##          lower     upper
## var1 0.1620258 0.3991629
## attr(,"Probability")
## [1] 0.95
```

Note that, while it is often claimed that Bayesian 95% credible intervals that do not cross zero can be used to indicate statistical significance in the classical (Frequentist) sense, this does not hold for variance components here as they are constrained to be positive in MCMCglmm. As such, a lower bound of the credible interval close to zero might actually indicate low confidence in a non-zero proportion of the phenotypic variance in exploration being explained by differences among individuals.

Let's do the same for boldness:

```
mcmc_prop_B <- mcmc_E_B_us$VCV[,"traitboldness:traitboldness.ID"]/(
  mcmc_E_B_us$VCV[,"traitboldness:traitboldness.ID"] +
    mcmc_E_B_us$VCV[,"traitboldness:traitboldness.units"]
)

mean(mcmc_prop_B)
```

```
## [1] 0.3751389
```

```
HPDinterval(mcmc_prop_B)
```

```
##          lower     upper
## var1 0.2602269 0.4977966
## attr(,"Probability")
## [1] 0.95
```

We can also use this process to estimate the mean and credible intervals of the correlation from our model (co)variances. We create a posterior distribution of the among-individual correlation by dividing the corresponding covariance between boldness and exploration by the product of the square root of their variances (i.e., standardising the covariance to a scale from -1 to 1):

```
mcmc_cor_EB <- mcmc_E_B_us$VCV[,"traitboldness:traitexploration.ID"]/
  (sqrt(mcmc_E_B_us$VCV[,"traitboldness:traitboldness.ID"])*
     sqrt(mcmc_E_B_us$VCV[,"traitexploration:traitexploration.ID"]))

plot(mcmc_cor_EB)
```

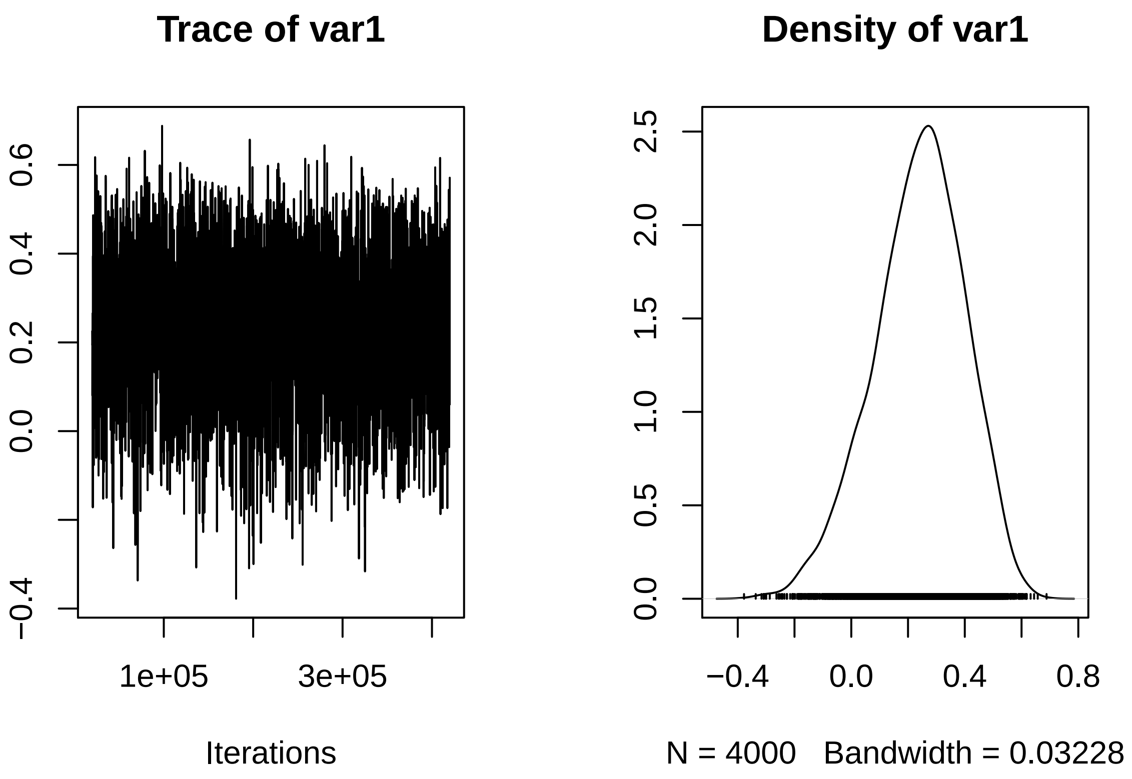


```r
mean(mcmc_cor_EB)
```

```
## [1] 0.2383352
```

```r
HPDinterval(mcmc_cor_EB)
```

```
##            lower    upper
## var1 -0.07829537 0.536206
## attr(,"Probability")
## [1] 0.95
```

In this case, because the correlation can take on either positive or negative then we can use the credible interval to assess statistical significance. Here the 95% credible interval spans zero, and since the model fit is good, we should conclude that there is no evidence of a statistically significant correlation.

To better demonstrate that BLUPs produce anticonservative hypothesis tests, we can plot the correlation estimates and their confidence/credible intervals from the two approaches that we have taken. The CI are taken directly from the `cor.test` function for the BLUPs, and for `MCMCglmm` they are taken from the posterior distribution of correlation samples (using the `HPDinterval` function).

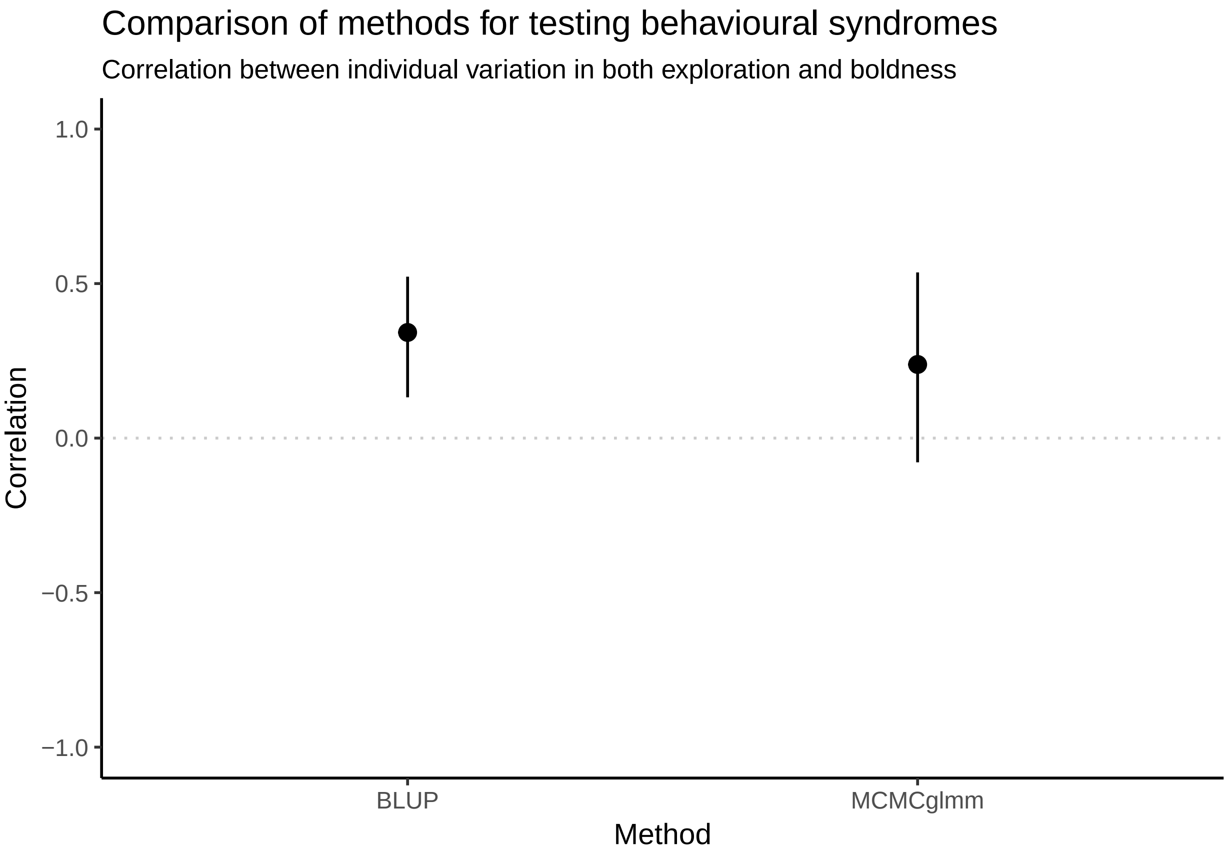

Here we can clearly see that the BLUPs method - having failed to carry through the error around the predictions of individual-level estimates - is anticonservative, with small confidence intervals (and a correspondingly small P-value, $P = 0.0019$). Testing the syndrome directly in a bivariate model that retains all the data, by comparison, enables us to capture the true uncertainty about the estimate of the correlation. This is reflected in the larger CI which, in this case, cross zero and thus indicate a lack of support for a statistically significant behavioural syndrome.

Adding further traits

As part of our data collection, we also have a single value of mating success for each individual (which we will use as a proxy for fitness). We are interested in how our personality traits correlate with variation in this fitness-related measure. While our test above showed that the correlation between the measured personality traits was not significant, there did appear to be some relationship — so we shall incorporate both personality traits and fitness into a single trivariate model for hypothesis testing.

In this case, because the new response variable to be added to our model is fitness, we are **not** going to mean-centre and scale by phenotypic standard deviations, but instead divide by the mean fitness value (such that we are investigating among-individual covariance between personality traits and **relative fitness**). We create this new variable, `rel_fitness`, as follows:

```
df_syndrome <- df_syndrome %>%
  mutate(rel_fitness = fitness/mean(fitness, na.rm=TRUE))
```

Note that we will refer to this relative fitness trait simply as 'fitness' below for simplicity's sake.

Setting up the model

Below, we will set up our main model, which will allow for heterogeneous among-individual variances in our 3 traits (boldness, exploration, fitness), and will estimate the covariance between them.

First, we set up a prior, which we specify in a similar way as the bivariate model. However, for the residuals (or 'within-individual' variance), we must bear in mind that we have only a single fitness value per individual — therefore, that trait has **no within-individual variance**, and **within-individual correlations involving fitness must be 0**. We can set the variance component to a particular value using the `fix` command, although as variances have to be positive we fix the within-individual variance in fitness to a small positive number (here, 0.0001):

```
prior_E_B_fit_1px = list(R = list(V = diag(c(1,1,0.0001),3,3), nu = 1.002, fix = 3),
                         G = list(G1 = list(V = diag(3), nu = 3,
                                            alpha.mu = rep(0,3),
                                            alpha.V = diag(25^2,3,3))))
```

Now, we can fit our model with these starting values and constraints. Again, we `cbind` our response variables on the left-hand side of the formula, and use `trait` to denote a multivariate model. We can also use the `at.level` keyword to specify that fixed effects are estimated only for certain traits — here, we test for an effect of assay repeat only on exploration and boldness (because these were measured repeatedly), while we test for the effect of body size on all of our traits.

Note that in the model specification below, we set the argument `pr = TRUE`. This saves the posterior distribution of the individual random effects (analagous to the BLUP from the REML analysis) so we can visualise them later, but does take up more memory (over 8Mb compared to <1Mb for a model run without saving these values).

Fit the model as follows (and be sure to use diagnostic checks). Note that I have increased the number of iterations (and both the burnin and thinning interval), so once it's underway, that's a good time to go and make a cup of tea... (the run will likely take over 20 minutes).

```
mcmc_E_B_fit <- MCMCglmm(cbind(scale(exploration),
                               scale(boldness),
                               rel_fitness) ~ trait-1 +
                           at.level(trait,1):scale(assay_rep, scale = FALSE) +
                           at.level(trait,2):scale(assay_rep, scale = FALSE) +
                           trait:scale(body_size),
                         random =~  us(trait):ID,
                         rcov =~ us(trait):units,
                         family = c("gaussian","gaussian","gaussian"),
                         prior = prior_E_B_fit_1px,
                         nitt=750000,
                         burnin=50000,
                         thin=175,
                         verbose = TRUE,
                         pr = TRUE,
                         data = as.data.frame(df_syndrome))
```

Take a look at the model summary:

```
summary(mcmc_E_B_fit)
```

As before, we get (co)variance estimates, credible intervals, and effective sample sizes for the among-individual and residual variance terms. Note that our constraint on the residual ('within-individual') variance term for our fitness measure: the `rel_fitness:rel_fitness.units` estimate is at 0.0001, with an effective sample

size of 0. You should also note that the within-individual covariance terms involving the fitness trait are very close to 0, with very small effective sample sizes, so the model has effectively not fit these covariances (which is what we wanted).

A quick sanity check also tells us that the correlation between boldness and exploration estimated in this model is the same as in our earlier bivariate model:

```
mcmc_E_B_fit_cor_EB <- mcmc_E_B_fit$VCV[,"traitboldness:traitexploration.ID"]/
  (sqrt(mcmc_E_B_fit$VCV[,"traitboldness:traitboldness.ID"])*
     sqrt(mcmc_E_B_fit$VCV[,"traitexploration:traitexploration.ID"]))

mean(mcmc_E_B_fit_cor_EB)
HPDinterval(mcmc_E_B_fit_cor_EB)
```

```
## [1] 0.2374761
##            lower     upper
## var1 -0.08700906 0.5379599
## attr(,"Probability")
## [1] 0.95
```

As before, we can use our posterior distributions to estimate the among-individual correlations between each of our traits of interest, and assess statistical significance using their 95% credible intervals from our `MCMCglmm` model:

```
mcmc_E_B_fit_cor_EB <- mcmc_E_B_fit$VCV[,"traitboldness:traitexploration.ID"]/
  (sqrt(mcmc_E_B_fit$VCV[,"traitboldness:traitboldness.ID"])*
     sqrt(mcmc_E_B_fit$VCV[,"traitexploration:traitexploration.ID"]))

mcmc_E_B_fit_cor_Efit <- mcmc_E_B_fit$VCV[,"traitrel_fitness:traitexploration.ID"]/
  (sqrt(mcmc_E_B_fit$VCV[,"traitrel_fitness:traitrel_fitness.ID"])*
     sqrt(mcmc_E_B_fit$VCV[,"traitexploration:traitexploration.ID"]))

mcmc_E_B_fit_cor_Bfit <- mcmc_E_B_fit$VCV[,"traitrel_fitness:traitboldness.ID"]/
  (sqrt(mcmc_E_B_fit$VCV[,"traitrel_fitness:traitrel_fitness.ID"])*
     sqrt(mcmc_E_B_fit$VCV[,"traitboldness:traitboldness.ID"]))

df_mcmc_cors <- data_frame(Traits = c("Exploration, Boldness",
                                      "Exploration, Fitness",
                                      "Boldness, Fitness"),
                           Estimate = c(mean(mcmc_E_B_fit_cor_EB),
                                        mean(mcmc_E_B_fit_cor_Efit),
                                        mean(mcmc_E_B_fit_cor_Bfit)),
                           Lower = c(HPDinterval(mcmc_E_B_fit_cor_EB)[,"lower"],
                                     HPDinterval(mcmc_E_B_fit_cor_Efit)[,"lower"],
                                     HPDinterval(mcmc_E_B_fit_cor_Bfit)[,"lower"]),
                           Upper = c(HPDinterval(mcmc_E_B_fit_cor_EB)[,"upper"],
                                     HPDinterval(mcmc_E_B_fit_cor_Efit)[,"upper"],
                                     HPDinterval(mcmc_E_B_fit_cor_Bfit)[,"upper"]))

ggplot(df_mcmc_cors, aes(x = Traits, y = Estimate)) +
  geom_pointrange(aes(ymin = Lower,
                      ymax = Upper)) +
  geom_hline(yintercept = 0,
             linetype = "dotted",
```

```
             alpha = 0.3) +
  scale_x_discrete(limits = c("Boldness, Fitness",
                              "Exploration, Fitness",
                              "Exploration, Boldness")) +
  labs(x = "Trait combinations",
       y = "Correlation (Estimate +/- 95% CIs)") +
  ylim(-1,1) +
  coord_flip() +
  theme_classic()
```

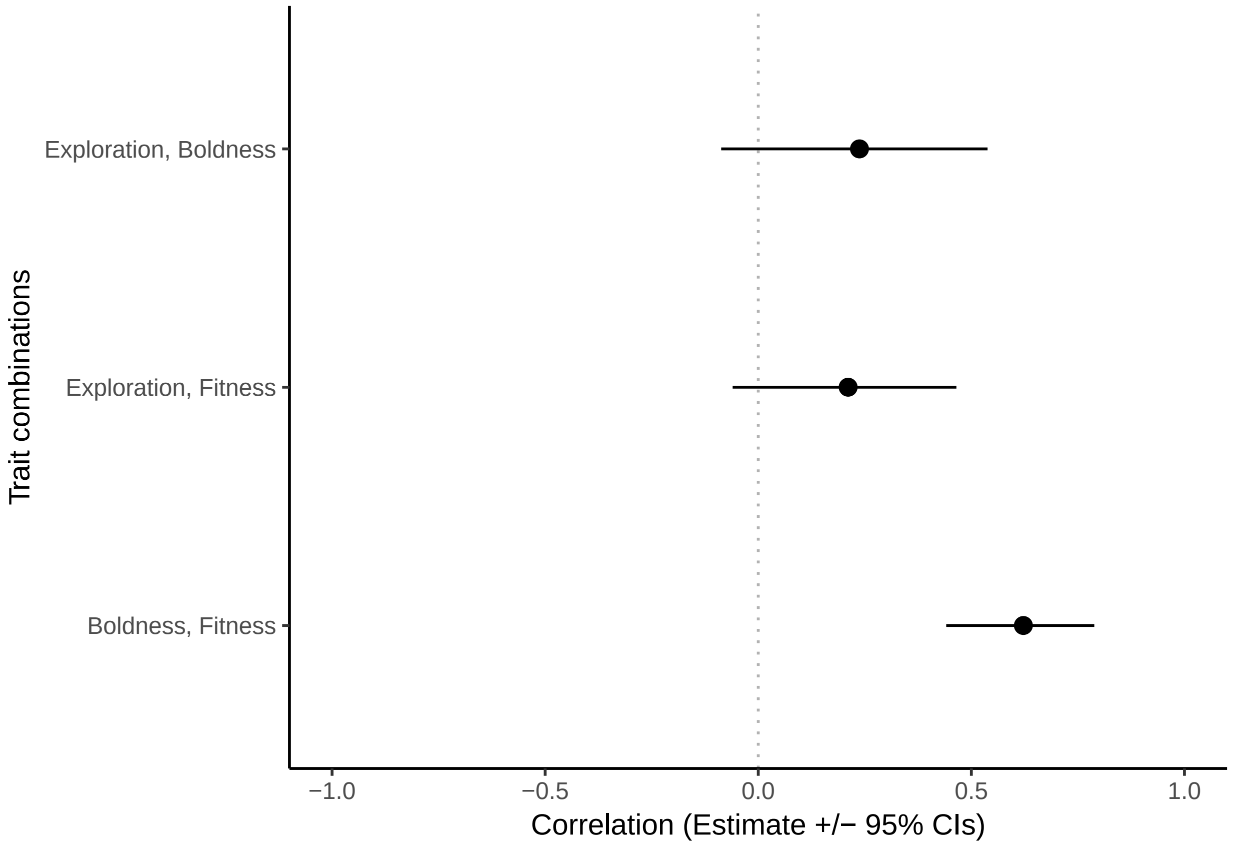

We might want to present our final results as a matrix with variances on the diagonals, covariances below and correlations above (with the lower and upper bounds of 95% CIs in parentheses):

	Exploration	Boldness	Fitness
Exploration	0.29<br>(0.13,0.45)	0.24<br>(-0.09,0.54)	0.21<br>(-0.06,0.46)
Boldness	0.08<br>(-0.04,0.21)	0.39<br>(0.22,0.57)	0.62<br>(0.44,0.79)
Fitness	0.03<br>(-0.01,0.07)	0.09<br>(0.05,0.14)	0.06<br>(0.04,0.08)

Conclusions

To conclude, then: we found that the correlation between boldness and exploration tends to be positive among male haggis, but this correlation is not statistically significant and thus does not provide strong evidence for a behavioural syndrome. However, inappropriate analysis of BLUP extracted from univariate models would lead to a different (erroneous) conclusion. We also found no statistically significant association between among-individual variation in exploration and fitness. However, we did find a statistically significant positive association between among-individual variation in boldness and our fitness proxy, indicating that bolder male haggis had greater mating success (see figure below).

Note: below, we use posterior modes of random effects (BLUPs from the MCMCglmm model) from our trivariate model to construct a figure that illustrates the association between boldness and fitness. Unlike its use in secondary statistical analyses, this is an appropriate use of BLUPs — i.e., just for illustrative purposes!

```
df_bf_coefs <- data_frame(Trait = attr(colMeans(mcmc_E_B_fit$Sol), "names"),
                          Value = colMeans(mcmc_E_B_fit$Sol)) %>%
  separate(Trait, c("Trait","Type","ID"), sep = "\\.", fill = "right") %>%
  filter(Type == "ID") %>%
  filter(Trait %in% c("traitboldness", "traitrel_fitness")) %>%
  select(-Type) %>%
  spread(Trait, Value)


# Find the regression line -
#  the covariance of boldness, relative fitness divided by
#  the variance in boldness
B_fit_slope <-  mcmc_E_B_fit$VCV[,"traitrel_fitness:traitboldness.ID"]/
  mcmc_E_B_fit$VCV[,"traitboldness:traitboldness.ID"]


ggplot(df_bf_coefs, aes(x = traitboldness, y = traitrel_fitness, group = ID)) +
  geom_point(alpha = 0.7) +
  geom_abline(intercept = 0, slope = mean(B_fit_slope)) +
  labs(x = "Boldness (BLUP)",
       y = "Relative fitness (BLUP)") +
  theme_classic()
```

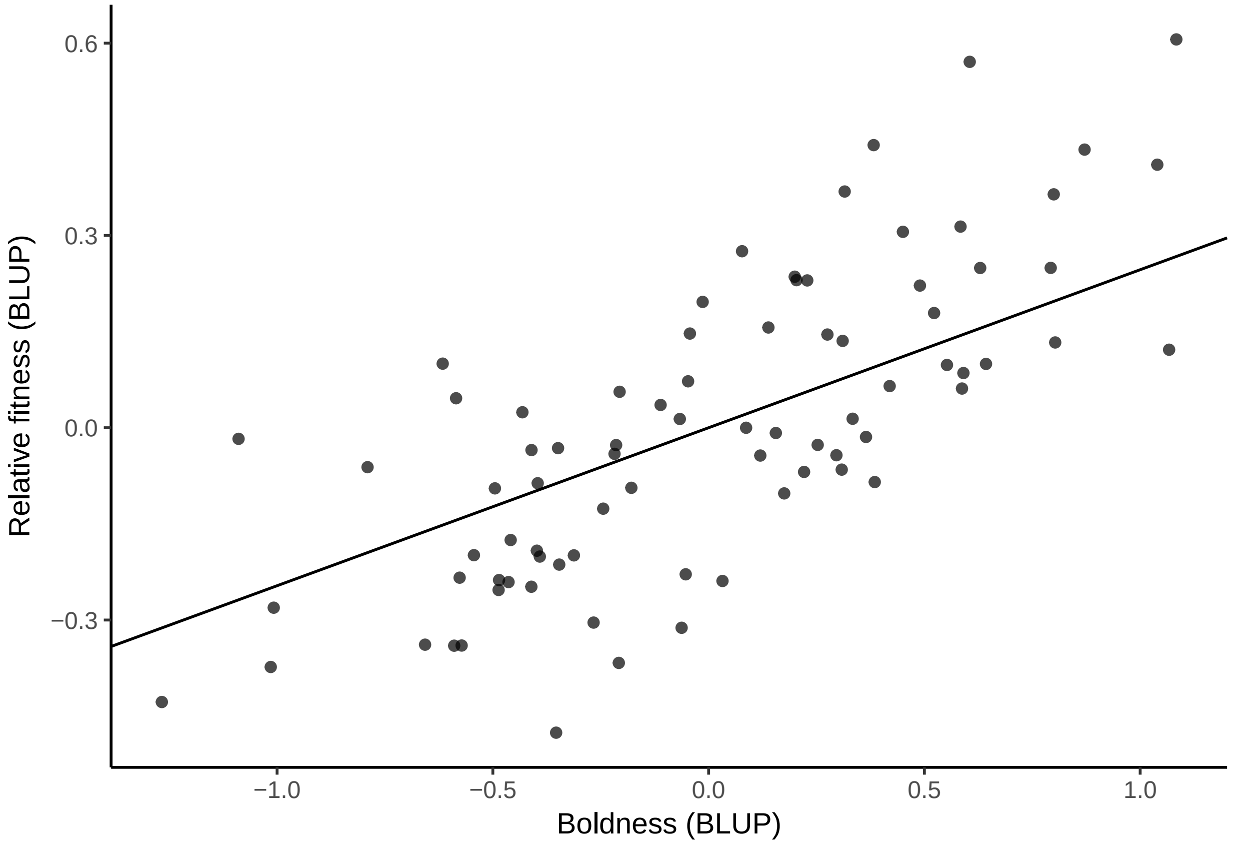

Further tutorials

We will continue to develop tutorials for multivariate modelling of individual (co)variation, which will cover some of the more advanced issues discussed in our paper. Please visit https://tomhouslay.com/tutorials/ for more information.
