# Supplementary material for: Avoiding the misuse of BLUP in behavioural ecology
Source: Behav Ecol. 2017 Mar 2;28(4):948–52. doi: 10.1093/beheco/arx023 (PMC5873244; doi:10.1093/beheco/arx023)
Supplement: IndivVar_MV_tutorial_ASReml [file arx023_suppl_indivvar_mv_tutorial_asreml.pdf]

Avoiding the misuse of BLUP in behavioral ecology: I. Multivariate modelling for individual variation (ASReml-R tutorial)

T.M. Houslay & A.J. Wilson, Behavioral Ecology

January 2017

Introduction

Overview

This tutorial accompanies our 2017 Behavioral Ecology paper, "Avoiding the misuse of BLUP in behavioral ecology". Below, we provide worked examples of multivariate statistical methods for directly testing hypotheses about associations between individual variation in behaviour and other traits. Below, we will:

- Test the correlation between two personality traits (behaviours measured repeatedly on individuals);
- Test for an association between these personality traits and a measure of fitness (one value per individual).

In this version, we illustrate these models using the `R` interface for `ASReml`, which is commercial software available from VSNi. We have provided a separate tutorial for the free `R` package `MCMCglmm`, but note that `MCMCglmm` uses Bayesian methods while `ASReml` uses maximum likelihood (and is therefore likely to be more familiar to users of the `R` package `lme4`).

Updates and further tutorials associated with this paper can be found at https://tomhouslay.com/tutorials/.

Aims

Please note that we do assume readers are familiar with the general principles of specifying univariate mixed effects models, and using diagnostic plots to check that the fitted model does not violate assumptions of the linear model. Readers unfamiliar with using univariate mixed effects models for modelling a single behavioural trait might prefer to start with (for example) Dingemanse & Dochtermann's 2013 paper, 'Quantifying individual variation in behaviour: mixed effects modelling approaches'.

We also use various methods for manipulating and visualising data frames using the `tidyverse` package (including `tidyr`, `dplyr`, `ggplot2` etc) — more details on their use can be found at http://r4ds.had.co.nz/.

In our tutorial, we aim to teach the following:

- How to phrase questions of interest in terms of variances and covariances (or derived correlations or regressions);
- How to incorporate more advanced model structures, such as:
  - Fixed effects that apply only to a subset of the response traits;
  - Traits which are measured a different number of times (*e.g.*, repeated measures of behaviour and a single value of breeding success);
- Hypothesis testing using likelihood ratio tests.

Packages required

There are several packages that you must have installed in `R` prior to starting this tutorial:

- `asreml` (note that this should be provided by the vendor, VSNi)
- `lme4`
- `nadiv`
- `tidyverse`
- `broom`

'Study system'

For this tutorial, we have collected data on a population of wild haggis (*Haggis scoticus*) that roam the Highlands of Scotland.

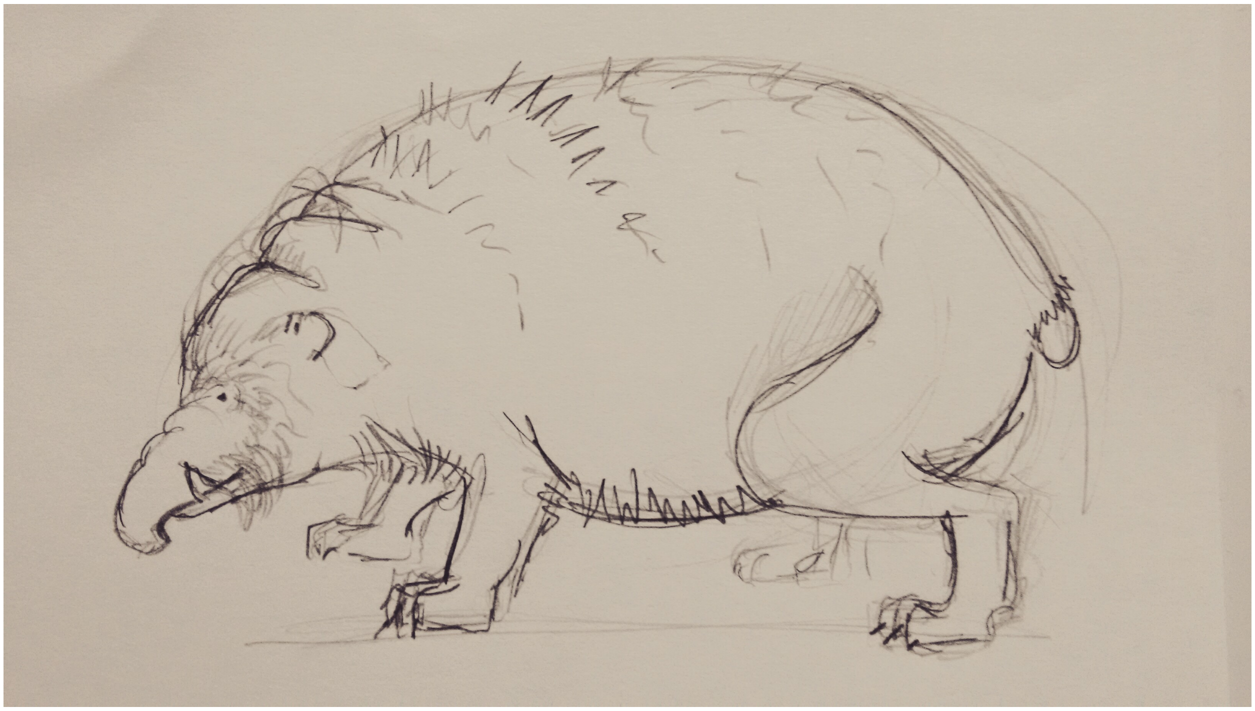

Figure 1: A male haggis in the wild (thanks to Emma Wood, http://www.ewood-art.co.uk/)

We tag all haggis individually when they emerge from their burrows as juveniles in their first spring. Here, we concentrate on male haggis, which are solitary and territorial. Previous work has identified behaviours that can be measured repeatedly, and used to represent the personality traits **boldness** and **exploration**. We also have the ability to collect a single measure of mating success (as a fitness proxy) for each male at the end of the season.

Behavioural syndromes

One type of 'behavioural syndrome' is a correlation between personality traits. Since personality can be viewed (under most definitions) as the repeatable (among-individual) component of behaviour, evidence

for the presence of a behavioural syndrome is provided by covariance among behaviours that arises from among-individual differences.

Here we have repeatedly measured behaviours that represent **boldness** and **exploration**. We observed each behaviour 4 times per individual. We also measured their body size on the day of behavioural assay so as to control for general size effects in our statistical models.

Load libraries and inspect data

```
library(lme4)
library(asreml)
library(tidyverse)
library(broom)
library(nadiv)

df_syndrome <- read_csv("syndrome.csv")
```

This data frame has 6 variables:

- Individual **ID**
- The repeat number for each behavioural test, **assay_rep**
- **boldness**, measured 4 times per individual
- **exploration**, measured 4 times per individual
- **fitness**, our measure of mating success, with a single value for each individual
- Individual **body_size**, as measured on the day of testing.

Univariate models

We first use the `R` package `lme4` to determine the proportion of phenotypic variation (adjusted for fixed effects) that is due to differences among individuals, separately for each behaviour. We assume readers have knowledge of these 'univariate' models and their use in behavioural studies — if not, there are various other publications that go into them in greater detail (e.g., Dingemanse & Dochtermann (2013)).

Boldness

Our model includes fixed effects of the assay repeat number (centred) and individual body size (centred and scaled to standard deviation units), as we wish to control for any systematic effects of these variables on individual behaviour. Please be aware that controlling variables are at your discretion — for example, while we want to characterise among-individual variance in boldness after controlling for size effects in this study, others may wish to characterise among-individual variance in boldness without such control. Indeed, using the techniques shown later in this tutorial, it would be entirely possible to characterise both among-individual variance in boldness and in size, and the among-individual covariance between these measurements.

```
lmer_b <- lmer(boldness ~ scale(assay_rep, scale=FALSE) +
                 scale(body_size) +
                 (1|ID),
               data = df_syndrome)
plot(lmer_b)
qqnorm(residuals(lmer_b))
hist(residuals(lmer_b))

summary(lmer_b)
```

```
## Linear mixed model fit by REML ['lmerMod']
## Formula: boldness ~ scale(assay_rep, scale = FALSE) + scale(body_size) +
##     (1 | ID)
##    Data: df_syndrome
##
## REML criterion at convergence: 1061.4
##
## Scaled residuals:
##     Min      1Q  Median      3Q     Max
## -2.3645 -0.6496 -0.1154  0.6463  2.6894
##
## Random effects:
##  Groups   Name        Variance Std.Dev.
##  ID       (Intercept) 0.6951   0.8337
##  Residual             1.1682   1.0808
## Number of obs: 320, groups:  ID, 80
##
## Fixed effects:
##                                 Estimate Std. Error t value
## (Intercept)                     20.09133    0.11108  180.87
## scale(assay_rep, scale = FALSE) -0.04805    0.05404   -0.89
## scale(body_size)                 0.14128    0.10893    1.30
##
## Correlation of Fixed Effects:
##             (Intr) s(_s=F
## s(_,s=FALSE  0.000
## scl(bdy_sz)  0.000 -0.002
```

Having examined diagnostic plots of the model fit, we can check the model `summary`. We are interested in the *random effects* section of the `lme4` model output (specifically the **variance** component — note that the standard deviation here is simply the square root of the variance). Evidence for 'animal personality' (or 'consistent among-individual differences in behaviour') in the literature is largely taken from the **repeatability** of behaviorual traits: we can compute this **repeatability** (also known as the *intraclass correlation coefficient*) by dividing the variance in the trait due to differences among individuals (V_{ID}) by the total phenotypic variance after accounting for the fixed effects ($V_{ID} + V_{residual}$). This can be done quickly and automatically through the use of the R package `broom`:

```
rep_bold <- tidy(lmer_b, effects = "ran_pars", scales = "vcov") %>%
  select(group, estimate) %>%
  spread(group, estimate) %>%
  mutate(repeatability = ID/(ID + Residual))

rep_bold
```

ID	Residual	repeatability
0.695	1.168	0.373

So we can see that 37.3% of the phenotypic variation in boldness (having controlled for body size and assay repeat number) is due to differences among individuals.

Let's do the same for our other behavioural trait, exploration:

Exploration

```
lmer_e <- lmer(exploration ~ scale(assay_rep, scale=FALSE) +
                 scale(body_size) +
                 (1|ID),
               data = df_syndrome)
```

```
rep_expl <- tidy(lmer_e, effects = "ran_pars", scales = "vcov") %>%
  select(group, estimate) %>%
  spread(group, estimate) %>%
  mutate(repeatability = ID/(ID + Residual))
```

ID	Residual	repeatability
0.362	0.909	0.285

Both of our traits of interest are repeatable at the among-individual level — the remaining question is characterising the association between these personality traits. Are individuals that are consistently bolder than average also more exploratory than average (and vice versa)?

Correlation using BLUPs

In our paper, we advise against the use of BLUPs due to their potential for spurious results due to anticonservative hypothesis tests and/or confidence intervals.

Here we will run through this method, purely so that we can then contrast the results with those that we get having (correctly) estimated the among-individual correlation between these behaviours directly from a multivariate model (in this case, bivariate).

We create two data frames of individual predictions extracted from model fits, one for each of our univariate `lme4` models for boldness and exploration. We then join these (by individual ID) to create a single data frame:

```
df_BLUPS_B <- data_frame(ID = row.names(ranef(lmer_b)$ID),
                         BLUP_B = ranef(lmer_b)$ID[,"(Intercept)"])

df_BLUPS_E <- data_frame(ID = row.names(ranef(lmer_e)$ID),
                         BLUP_E = ranef(lmer_e)$ID[,"(Intercept)"])

df_BLUPS_EB <- left_join(df_BLUPS_E,
                         df_BLUPS_B,
                         by = "ID")
```

We can plot these to see what our expectation of a correlation might be:

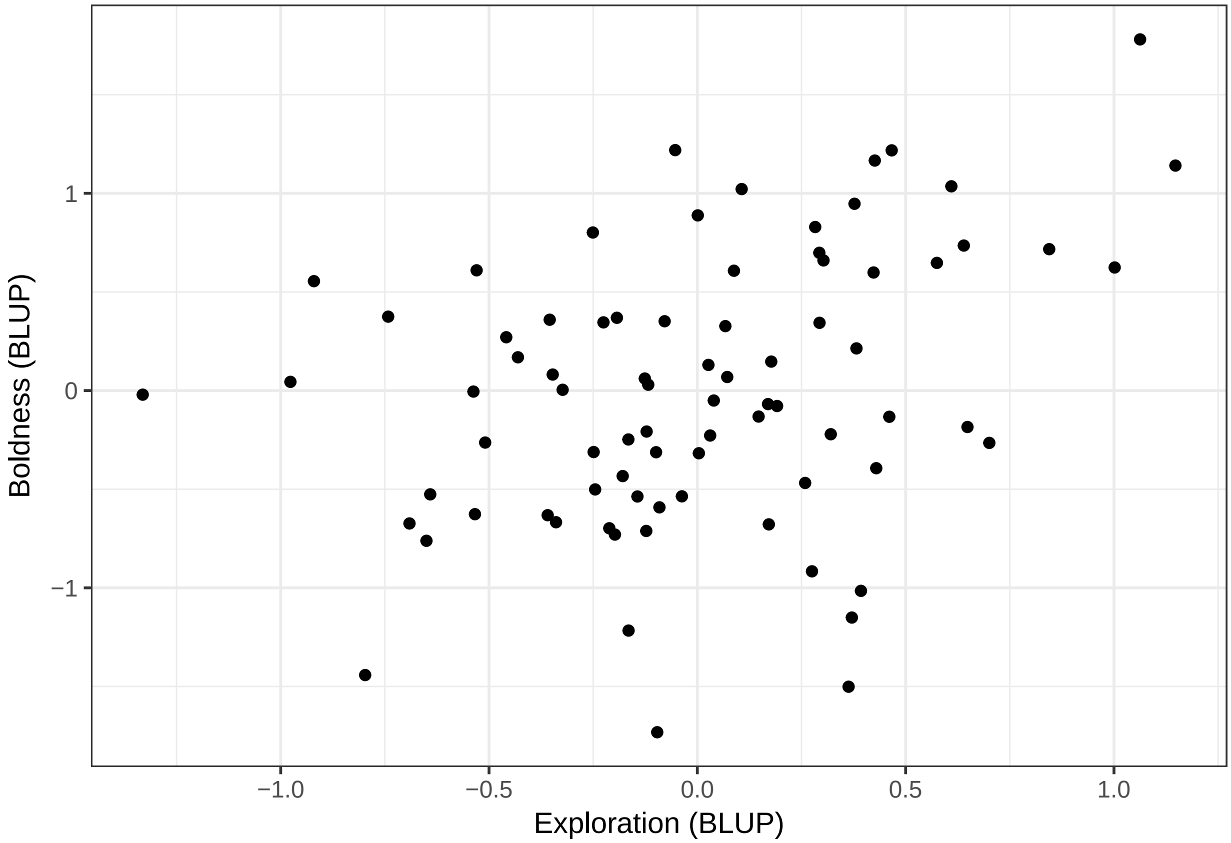

..and then simply perform a correlation test of these two traits using the `cor.test` function:

```
cor.test(df_BLUPS_EB$BLUP_E,
         df_BLUPS_EB$BLUP_B)
```

```
##
##  Pearson's product-moment correlation
##
## data:  df_BLUPS_EB$BLUP_E and df_BLUPS_EB$BLUP_B
## t = 3.2131, df = 78, p-value = 0.00191
## alternative hypothesis: true correlation is not equal to 0
## 95 percent confidence interval:
##  0.1320924 0.5223645
## sample estimates:
##       cor
## 0.3418867
```

As you can see, we get a positive correlation with a very small p-value ($P = 0.0019$), indicating that these traits are involved in a behavioural syndrome. While the correlation itself is fairly weak ($r = 0.34$), it appears to be highly significant, and suggests that individuals that are bolder than average also tend to be more exploratory than average.

However, as discussed in our paper (and in greater detail by Hadfield *et al*), using BLUPs in this way leads to anticonservative significance tests. This is because the error inherent in their prediction is not carried forward from the `lmer` models to the subsequent analysis (in this case, a correlation test). To illustrate this point quickly, below we plot the individual estimates along with their associated standard errors:

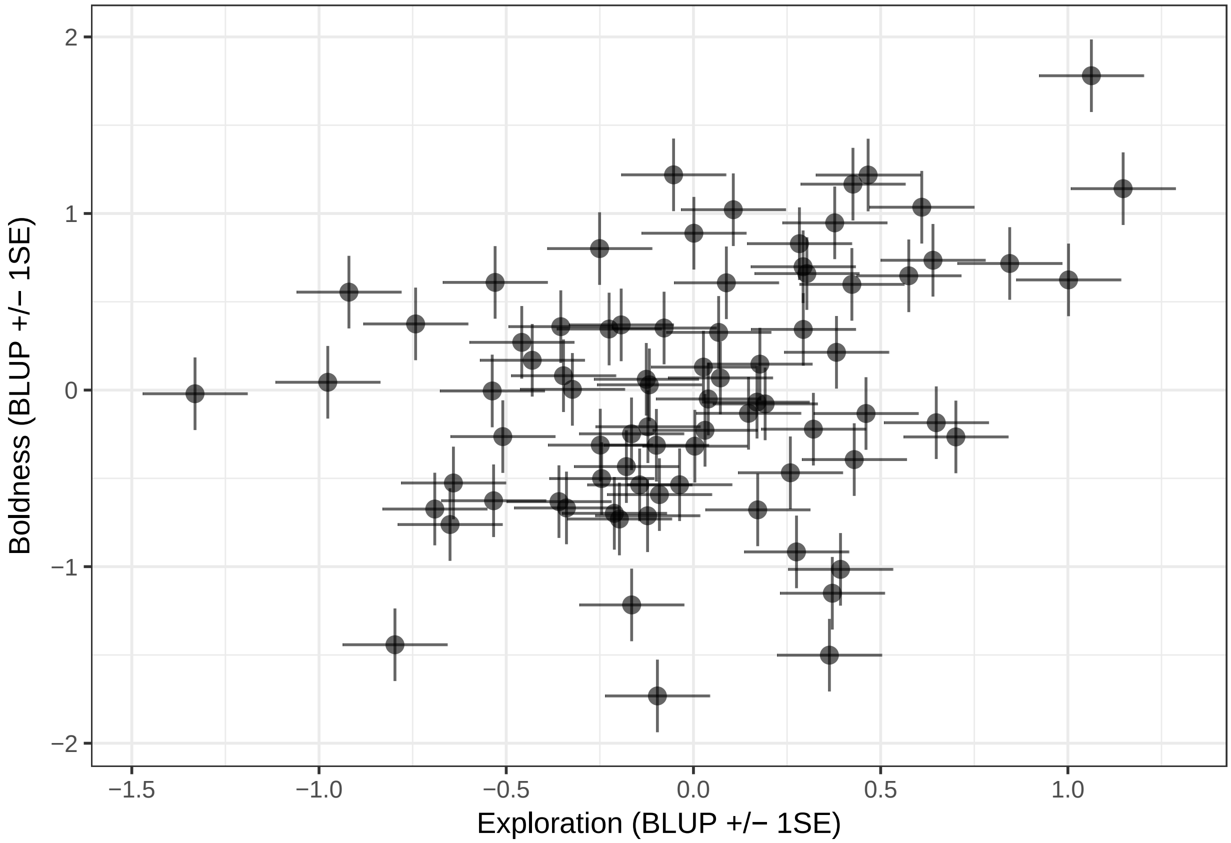

We now go on to estimate the correlation between these behaviours directly in a multivariate model, using `ASreml`.

Bivariate models

The correct approach for testing the hypothesised behavioural syndrome uses both response variables in a two-trait ('bivariate') mixed model. This model estimates the among-individual variance for each response variable (and the covariance between them). Separate (co)variances are also fitted for the residual variation. The bivariate model also allows for fixed effects to be fitted on both response variables.

We set up our model using the `asreml` function call, with our bivariate response variable being **exploration** and **boldness** bound together using `cbind`. You will also note that we `scale` our response variables, meaning that each is centred at their mean value and standardised to units of 1 standard deviation. This is not essential, but simply makes it easier for the model to be fit. Scaling the response variables also aids our understanding of the output, as both boldness and exploration are now on the same scale.

```
asr_E_B_us <- asreml(cbind(scale(exploration),
                           scale(boldness)) ~ trait +
                       trait:scale(assay_rep, scale = FALSE) +
                       trait:scale(body_size),
                     random =~ ID:us(trait, init = c(1,
                                                     0.1,1)),
                     rcov =~ units:us(trait, init = c(0.1,
                                                      0.1,0.1)),
                     data = df_syndrome,
                     maxiter = 100)
```

On the right hand side of our model formula, we use the `trait` keyword to specify that this is a multivariate model — `trait` itself tells the model to give us the intercept for each trait. We then interact `trait` with our fixed effects, **assay__rep** and **body__size**, so that we get estimates for the effect of these variables on each of our behaviours.

Our random effects structure starts with the `random` effects, where we tell the model to fit an 'unstructured' (`us`) covariance matrix for the grouping variable **ID**. This means that we want to calculate the variance in exploration due to differences among individuals, the variance in boldness due to differences among individuals, and the **covariance** between these variances.

Next, we set a structure for the residual variation (`rcov`), which is also sometimes known as the 'within-individual variation'. As we have repeated measures for both traits at the individual level, we also set an unstructured covariance matrix, which finds the residual variance for each trait and also allows the residuals to covary across the two traits.

Finally, we provide the name of the data frame, and a maximum number of iterations for `ASReml` to attempt to fit the model.

After the model has been fit by `ASReml`, we can check the fit using the same type of model diagnostic plots as we use for `lme4`:

```
plot(residuals(asr_E_B_us)~fitted(asr_E_B_us))
qqnorm(residuals(asr_E_B_us))
hist(residuals(asr_E_B_us))
```

The `summary` part of the `ASReml` model fit contains a large amount of information, so it is best to look only at certain parts of it at a single time. While we are not particularly interested in the fixed effects for current purposes, you can inspect these using the following code to check whether there were any large effects of assay repeat or body size on either trait:

```
summary(asr_E_B_us, all=T)$coef.fixed
```

We can see that there is a separate intercept for both personality traits (no surprise that these are very close to zero, given that we mean-centred and scaled each trait before fitting the model), and an estimate of the effect of assay repeat and body size on both traits. None of these appear to be large effects, so let's move on to the more interesting parts — the random effects estimates:

```
summary(asr_E_B_us)$varcomp
```

```
##                                              gamma component  std.error
## ID:trait!trait.exploration:exploration 0.2863101 0.2863101 0.07637361
## ID:trait!trait.boldness:exploration    0.0883864 0.0883864 0.06067166
## ID:trait!trait.boldness:boldness       0.3733306 0.3733306 0.08607573
## R!variance                             1.0000000 1.0000000         NA
## R!trait.exploration:exploration        0.7184419 0.7184419 0.06572786
## R!trait.boldness:exploration           0.3263211 0.3263211 0.04829180
## R!trait.boldness:boldness              0.6274169 0.6274169 0.05740290
##                                          z.ratio constraint
## ID:trait!trait.exploration:exploration  3.748810   Positive
## ID:trait!trait.boldness:exploration     1.456799   Positive
## ID:trait!trait.boldness:boldness        4.337234   Positive
## R!variance                                    NA      Fixed
## R!trait.exploration:exploration        10.930554   Positive
## R!trait.boldness:exploration            6.757279   Positive
## R!trait.boldness:boldness              10.930055   Positive
```

In the above summary table, we have the among-individual (co)variances listed first (starting with `ID`), then the residual (or within-individual) (co)variances (starting with `R`). You will notice that the variance estimates here are actually close to the `lme4` repeatability estimates, because our response variables were scaled to phenotypic standard deviations. We can also find the 'adjusted repeatability' (i.e., the repeatability conditional on the fixed effects) for each trait by dividing its among-individual variance estimate by the sum of its among-individual and residual variances.

Here, we use the `pin` function from the `nadiv` package (Wolak 2012) to estimate the repeatability and its standard error for each trait, conditional on the effects of assay repeat and body size. For this function, we provide the name of the model object, followed by a name that we want to give the estimate being returned, and a formula for the calculation. Each 'V' term in the formula refers to a variance component, using its position in the model summary shown above.

```
nadiv:::pin(asr_E_B_us, prop_expl ~ V1/(V1+V5))
nadiv:::pin(asr_E_B_us, prop_bold ~ V3/(V3+V7))
```

```
##           Estimate         SE
## prop_expl 0.284956 0.06113612
##            Estimate         SE
## prop_bold 0.3730518 0.06124283
```

We can also use this function to calculate the estimate and standard error of the correlation from our model (co)variances. We do this by specifying the formula for the correlation:

```
nadiv:::pin(asr_E_B_us, cor ~ V2/(sqrt(V1)*sqrt(V3)))
```

```
##      Estimate        SE
## cor 0.2703462 0.1594158
```

In this case, the estimate is similar (here, slightly lower) than our correlation estimate using BLUPs. However, if we consider confidence intervals as +/- 1.96SE around the estimate, the lower bound of the confidence interval would actually be -0.042. **With confidence intervals straddling zero, we would conclude that this correlation is likely non-significant**. As the use of standard errors in this way is only approximate, we should also test our hypothesis formally using likelihood ratio tests.

Hypothesis testing

We can now test the statistical significance of this correlation directly, by fitting a second model without the among-individual covariance between our two behavioural traits, and then using a likelihood ratio test to determine whether the model with the covariance produces a better fit.

Here, we use the `idh` structure for our random effects. This stands for 'identity matrix' (i.e., with 0s on the off-diagonals) with heterogeneous variances (i.e., the variance components for our two response traits are allowed to be different from one another). The rest of the model is identical to the `us` version.

```
asr_E_B_idh <- asreml(cbind(scale(exploration),
                            scale(boldness)) ~ trait +
                        trait:scale(assay_rep, scale = FALSE) +
                        trait:scale(body_size),
                      random =~ ID:idh(trait, init = c(1,1)),
                      rcov =~ units:us(trait, init = c(0.1,
                                                       0.1,0.1)),
                      data = df_syndrome,
                      maxiter = 100)
```

The likelihood ratio test is calculated as twice the difference between model log-likelihoods, on a single degree of freedom (the covariance term):

```
pchisq(2*(asr_E_B_us$loglik - asr_E_B_idh$loglik),
          1, lower.tail = FALSE)
```

```
## [1] 0.1170403
```

In sharp contrast to the highly-significant P-value given by a correlation test using BLUPs, here we find **no evidence for a behavioural syndrome between exploration and boldness**.

To better understand why BLUPs produce an anticonservative p-value in comparison to multivariate models, we should plot the correlation estimates and their confidence intervals. The confidence intervals are taken directly from the `cor.test` function for BLUPs, and for `ASReml` they are calculated as 1.96 times the standard error from the `pin` function.

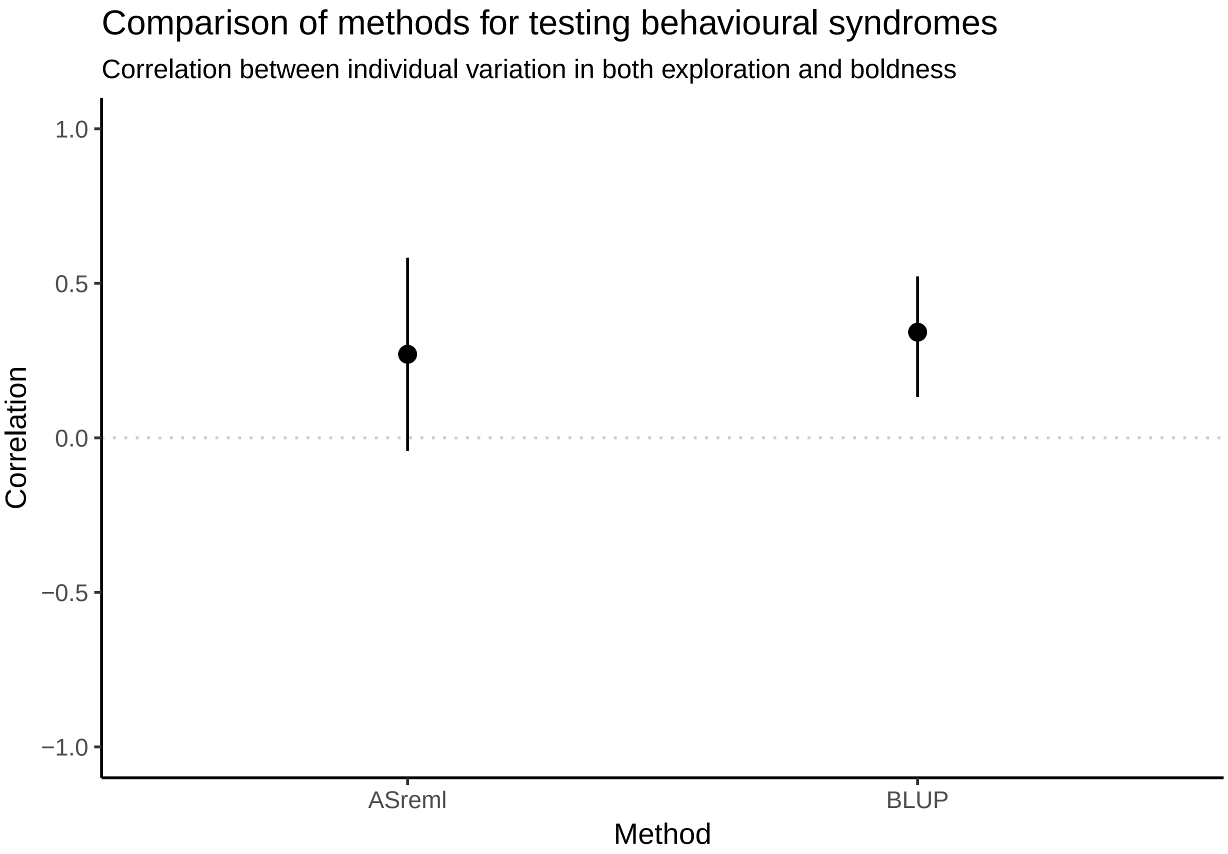

Here we can clearly see that the BLUPs method - having failed to carry through the error around the predictions of individual-level estimates - is anticonservative, with small confidence intervals and a correspondingly small P-value ($P = 0.0019$). Testing the syndrome directly in a bivariate model that retains all the data, by comparison, enables us to capture the true uncertainty about the estimate of the correlation. This is reflected in the larger confidence intervals and, in this case, the non-significant P-value ($P = 0.117$).

Adding further traits

As part of our data collection, we also have a single value of mating success for each individual (which we will use as a proxy for fitness). We are interested in whether our personality traits are associated with variation

in this fitness-related measure. While our test above showed that the correlation between the measured personality traits was not significant, there did appear to be some relationship — so we shall incorporate both personality traits and fitness into a single trivariate model for hypothesis testing.

In this case, because the new response variable to be added to our model is fitness, we are **not** going to mean-centre and scale by phenotypic standard deviations, but instead divide by the mean fitness value (such that we are investigating among-individual covariance between personality traits and **relative fitness**). We create this new variable, `rel_fitness`, as follows:

```
df_syndrome <- df_syndrome %>%
  mutate(rel_fitness = fitness/mean(fitness, na.rm=TRUE))
```

Note that we will refer to this relative fitness trait simply as 'fitness' below for simplicity's sake.

Setting up the model

Below, we will set up our main model, which will allow for heterogeneous among-individual variances in our 3 traits (boldness, exploration, fitness), and will estimate the associations between them. Note, however, that we will use the `corgh` structure instead of `us` in the random effects. These structures fit the same model, but on a correlation rather than covariance scale. Note in this case we are just using `corgh` because it makes it easier in `ASReml` to specify some constraints that we require and (as we will see later, we can always backcalculate the covariances from the estimated correlations if we want them).

First, we set up starting values from the model, which we also use to set some constraints. We set constraints in `ASReml` by specifying some starting values in a numeric vector, then giving each value a 'name' that corresponds to how `ASReml` should treat the corresponding part of the random effects matrix during model fitting:

- U: Unconstrained (can take any value, positive or negative)
- P: Positive (must be a positive value)
- F: Fixed (remains fixed at the given value)

An important point: while the starting values (`init`) for the `us` structure were provided in the form of the lower triangle of a covariance matrix, for `corgh` we provide the correlations first, and then the variances.

For the random effects, we set generic starting values — the 3 correlations have starting values close to 0 and are unconstrained, while the variance components have starting values of unit variance (and are constrained to be positive values):

```
init_E_B_fit_cor <- c(0.1,
                      0.1,0.1,
                      1,1,1)
names(init_E_B_fit_cor) <- c("U",
                             "U","U",
                             "P","P","P")
```

For the residuals (or 'within-individual' variance), we must bear in mind that we have only a single fitness value per individual — therefore, that trait has **no within-individual variance**, and **within-individual correlations involving fitness must be set to zero as they cannot be estimated**. We set the starting value for both correlations to 0 below, and denote them as fixed at those values using 'F'. The variance component is slightly trickier — variances have to be positive, therefore we simply fix the within-individual variance at a very small positive number (here, 1e-08 — i.e., so small as to be effectively 0):

```
init_E_B_fit_res <- c(0.1,
                      0,0,
                      0.1, 0.1, 1e-08)
names(init_E_B_fit_res) <- c("U",
                             "F","F",
                             "P","P","F")
```

Now, we can fit our model with these starting values and constraints. Again, we `cbind` our response variables on the left-hand side of the formula, and use `trait` to denote a multivariate model. Remember that we have created the 'relative fitness' variable by essentially scaling by its mean, so this does not need to be scaled as the behavioural traits are.

We can also use the `at` keyword to specify that fixed effects are estimated only for certain traits — here, we test for an effect of assay repeat only on exploration and boldness (because these were measured repeatedly), while we test for the effect of body size on all of our traits.

Fit the model as follows (and be sure to use visual diagnostic checks of the residuals):

```
asr_E_B_fit_cor <- asreml(cbind(scale(exploration),
                                scale(boldness),
                                rel_fitness) ~ trait +
                            at(trait,1):assay_rep +
                            at(trait,2):assay_rep +
                            trait:scale(body_size),
                          random =~ ID:corgh(trait, init = init_E_B_fit_cor),
                          rcov =~ units:corgh(trait, init = init_E_B_fit_res),
                          data = df_syndrome,
                          maxiter = 500)
```

We can take a quick look at the fixed effects:

```
summary(asr_E_B_fit_cor, all=T)$coef.fixed
```

Below, we specify that we want to look at the variance components using `$varcomp`. In the interests of space, we will request only the `component` (i.e., the variance estimate) and its `std.error`:

```
summary(asr_E_B_fit_cor)$varcomp[,c("component","std.error")]
```

```
##                                                  component   std.error
## ID:trait!trait.boldness:!trait.exploration.cor    0.27031497 0.159419988
## ID:trait!trait.rel_fitness:!trait.exploration.cor 0.23386699 0.138687881
## ID:trait!trait.rel_fitness:!trait.boldness.cor    0.66168293 0.087961997
## ID:trait!trait.exploration                        0.28630613 0.076372770
## ID:trait!trait.boldness                           0.37322016 0.086051330
## ID:trait!trait.rel_fitness                        0.05659086 0.009060437
## R!variance                                        1.00000000          NA
## R!trait.boldness:!trait.exploration.cor           0.48603894 0.049410253
## R!trait.rel_fitness:!trait.exploration.cor        0.00000000          NA
## R!trait.rel_fitness:!trait.boldness.cor           0.00000000          NA
## R!trait.exploration                               0.71844420 0.065728071
## R!trait.boldness                                  0.62744922 0.057405898
## R!trait.rel_fitness                               0.00000001          NA
```

Here we can see that the fit provides us with estimates and standard errors of:

- 3 among-individual correlations;
- 3 among-individual variance components;
- 3 within-individual correlations;
- 3 within-individual variance components.

You can see from the estimates that our constraints have worked in the model: within-individual correlations featuring fitness are at 0, and the residual fitness variance is a very small positive number (such that all the variation is at the among-individual level).

A quick sanity check also tells us that the correlation between boldness and exploration (the first variance component in our summary table above, $r = 0.27$ SE 0.159) estimated in this model is the same as in our earlier bivariate model.

From a first glance at the correlation estimates and their associated standard errors, it appears likely that there is a significant among-individual correlation between relative fitness and boldness ($r = 0.662$ SE 0.088), but not between relative fitness and exploration ($r = 0.234$ SE 0.139).

Hypothesis testing

We can again use likelihood ratio tests for hypothesis testing with these models. We first test for an association between relative fitness and our bivariate personality phenotype (defined by the two traits). We do this by fixing both correlations with fitness ($r_{\text{boldness,fitness}}$ and $r_{\text{exploration,fitness}}$) to 0. We then use a likelihood ratio test to analytically compare our main model (with all correlations estimated) to this second model (no correlation between fitness and boldness/exploration), which tests whether allowing those correlations provides a statistically significant improvement in the model fit. Note this is not testing the significance of each trait-fitness correlation separately, it is testing whether there is any significant fitness-phenotype correlation overall.

We set the correlations to 0 as follows:

```
init_E_B_fit_cor_FEB0 <- c(0.1,
                           0,0,
                           1,1,1)
names(init_E_B_fit_cor_FEB0) <- c("U",
                                  "F","F",
                                  "P","P","P")

asr_E_B_fit_cor_FEB0 <- asreml(cbind(scale(exploration),
                                     scale(boldness),
                                     rel_fitness) ~ trait +
                                 at(trait,1):assay_rep +
                                 at(trait,2):assay_rep +
                                 trait:scale(body_size),
                               random =~ ID:corgh(trait, init = init_E_B_fit_cor_FEB0),
                               rcov =~ units:corgh(trait, init = init_E_B_fit_res),
                               data = df_syndrome,
                               maxiter = 800)
```

We then test the difference in model fits using a likelihood ratio test with 2 degrees of freedom:

```
pchisq(2*(asr_E_B_fit_cor$loglik - asr_E_B_fit_cor_FEB0$loglik),
           2, lower.tail = FALSE)
```

```
## [1] 5.654352e-07
```

Here we find evidence of significant correlation structure — based on the estimates and SEs from the model summary, it's a fairly safe bet that this is being driven by the fitness-boldness association. If tests of each of the specfic trait-fitness correlations are needed, we advise using pairwise models (but note of course that multiple testing issues might require consideration if you want to statistically test every pairwise correlation estimate and you have a lot of traits). We will fit the two bivariate trait-fitness models below for completeness, and they should confirm our suspicions about which personality trait is driving the correlation between the bivariate behavioural phenotype and fitness.

As with tests of the earlier bivariate models for behavioural syndromes, we fit models with both `us` and `idh` structures (or `corgh` with setting the correlation to 0) for hypothesis testing using likelihood ratio tests. In this case, we also have to set the residual variation in fitness to a very small (near-zero) positive number, and we do not fit a residual covariance. Here we demonstrate for boldness and fitness:

```
init_fitbiv_res <- c(0.1,1e-08)
names(init_fitbiv_res) <- c("P","F")

asr_B_fit_us <- asreml(cbind(scale(boldness),
                             rel_fitness) ~ trait +
                         at(trait,1):assay_rep +
                         trait:scale(body_size),
                       random =~ ID:us(trait, init = c(1,
                                                       0.1,1)),
                       rcov =~ units:idh(trait, init = init_fitbiv_res),
                       data = df_syndrome,
                       maxiter = 800)

asr_B_fit_idh <- asreml(cbind(scale(boldness),
                              rel_fitness) ~ trait +
                          at(trait,1):assay_rep +
                          trait:scale(body_size),
                        random =~ ID:idh(trait, init = c(1,1)),
                        rcov =~ units:idh(trait, init = init_fitbiv_res),
                        data = df_syndrome,
                        maxiter = 800)
```

```
## [1] 8.164003e-08
```

We can now run the same test for exploration and fitness:

```
## [1] 0.1024701
```

As we had anticipated from the estimate and standard error of the correlations in our trivariate model, the association between individual variation in boldness and relative fitness is significant, while there is no evidence for a significant association between individual variation in exploration and fitness.

A slight digression: converting correlations back to covariances can be useful

While we set up the trivariate model to output results in terms of correlation matrices, we could have fit the model on a covariance scale using `us`. While correlations are intuitive, sometimes having the answers on the covariance scale is useful. For instance, in the current example, the trait-fitness correlations could be used to infer selection — but if we wanted to express the strength of that selection, the normal way to do so is through selection differentials. These are the trait – (relative) fitness covariances, and/or selection gradients (the partial regressions of relative fitness on traits which can be calculated from variance and covariance terms).

Since a correlation is simply the covariance rescaled by the product of the squared variances, we can retrieve the covariance terms by simply rearranging as follows:

$$COV_{T1,T2} = r_{T1,T2} \times \sqrt{V_{T1}} \times \sqrt{V_{T2}}$$

Again, the `pin` function comes to our rescue. As an example, we can get the covariance between exploration and boldness from our trivariate model (with `corgh` correlation-structure) as follows:

```
nadiv:::pin(asr_E_B_fit_cor, cov_E_B ~ V1*sqrt(V4)*sqrt(V5))
```

```
##           Estimate         SE
## cov_E_B 0.08836249 0.06066255
```

We might want to present our final results as a matrix with variances on the diagonals, covariances below and correlations above (with standard errors in parentheses):

	Exploration	Boldness	Fitness
Exploration	0.29 (0.08)	0.27 (0.16)	0.23 (0.14)
Boldness	0.09 (0.06)	0.37 (0.09)	0.66 (0.09)
Fitness	0.03 (0.02)	0.1 (0.02)	0.06 (0.01)

Conclusions

To conclude, then: we found that the correlation between boldness and exploration tends to be positive among male haggis. This correlation is not statistically significant, and thus does not provide strong evidence for a behavioural syndrome. However, inappropriate analysis of BLUP extracted from univariate models would lead to a different (erroneous) conclusion. We also found no statistically significant association between among-individual variation in exploration and fitness. However, we did find a statistically significant positive association between among-individual variation in boldness and our fitness proxy, indicating that bolder male haggis had greater mating success (see figure below).

Note: below, we use BLUPs from our trivariate model to construct a figure that illustrates the association between boldness and fitness. Unlike its use in secondary statistical analyses, this is an appropriate use of BLUPs — i.e., just for illustrative purposes!

```
# Retrieve BLUPs from ASReml trivariate model
#  and reform into data frame for plotting
df_bf_coefs <- data_frame(Trait = attr(asr_E_B_fit_cor$coefficients$random, "names"),
                          Value = asr_E_B_fit_cor$coefficients$random) %>%
  separate(Trait, c("ID","Trait"), sep = ":") %>%
  filter(Trait %in% c("trait_boldness", "trait_rel_fitness")) %>%
  spread(Trait, Value)
```

```
# Find the regression line -
#  the covariance of boldness, relative fitness divided by
#  the variance in boldness
B_fit_slope <-  as.numeric(nadiv:::pin(asr_E_B_fit_cor,
                                        slope ~ (V3*sqrt(V5)*sqrt(V6))/
                                          V5)$Estimate)

ggplot(df_bf_coefs, aes(x = trait_boldness, y = trait_rel_fitness, group = ID)) +
  geom_point(alpha = 0.7) +
  geom_abline(intercept = 0, slope = B_fit_slope) +
  labs(x = "Boldness (BLUP)",
       y = "Relative fitness (BLUP)") +
  theme_classic()
```

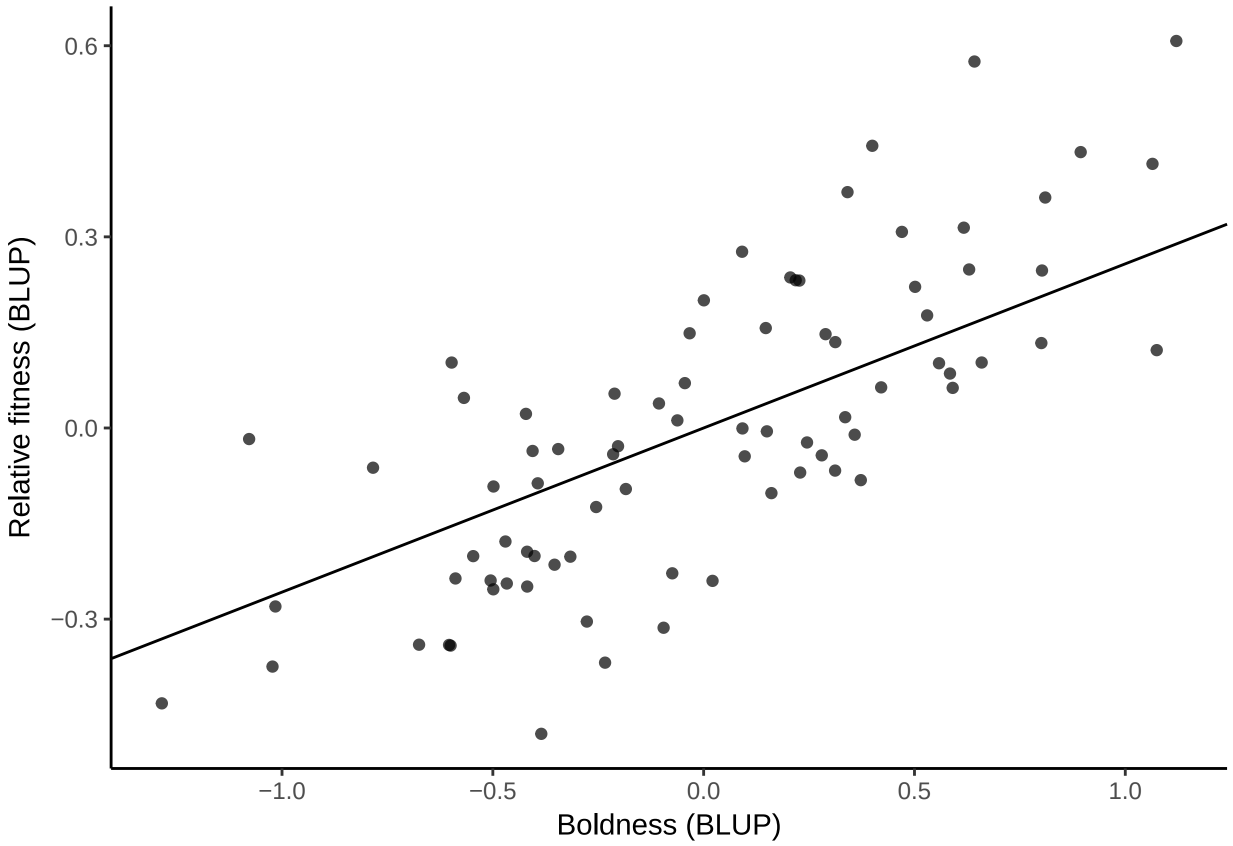

Further tutorials

We will continue to develop tutorials for multivariate modelling of individual (co)variation, which will cover some of the more advanced issues discussed in our paper. Please visit https://tomhouslay.com/tutorials/ for more information.
